# Biological soil crusts on agricultural soils of mesic regions promote microbial cross-kingdom co-occurrences and nutrient retention

**DOI:** 10.3389/fmicb.2023.1169958

**Published:** 2023-07-14

**Authors:** Julia Katharina Kurth, Martin Albrecht, Karin Glaser, Ulf Karsten, Gisle Vestergaard, Martin Armbruster, Susanne Kublik, Christoph A. O. Schmid, Michael Schloter, Stefanie Schulz

**Affiliations:** ^1^Chair for Environmental Microbiology, TUM School of Life Science, Technical University Munich, Freising, Germany; ^2^Environmental Health Centre, Research Unit for Comparative Microbiome Analysis, Helmholtz Zentrum München - Deutsches Forschungszentrum für Gesundheit und Umwelt, Neuherberg, Germany; ^3^Department of Applied Ecology and Phycology, Institute of Biological Sciences, University of Rostock, Rostock, Germany; ^4^Section of Microbiology, Department of Biology, University of Copenhagen, Copenhagen, Denmark; ^5^Agricultural Analytical and Research Institute Speyer (LUFA Speyer), Speyer, Germany

**Keywords:** biocrust, amplicon, hotspot, managed site, Cyanobacteria, microbial co-occurrence network, mesophilic region

## Abstract

**Introduction:**

Biological soil crusts (biocrusts) are known as biological hotspots on undisturbed, nutrient-poor bare soil surfaces and until now, are mostly observed in (semi-) arid regions but are currently poorly understood in agricultural systems. This is a crucial knowledge gap because managed sites of mesic regions can quickly cover large areas. Thus, we addressed the questions (i) if biocrusts from agricultural sites of mesic regions also increase nutrients and microbial biomass as their (semi-) arid counterparts, and (ii) how microbial community assemblage in those biocrusts is influenced by disturbances like different fertilization and tillage regimes.

**Methods:**

We compared phototrophic biomass, nutrient concentrations as well as the abundance, diversity and co-occurrence of Archaea, Bacteria, and Fungi in biocrusts and bare soils at a site with low agricultural soil quality.

**Results and Discussion:**

Biocrusts built up significant quantities of phototrophic and microbial biomass and stored more nutrients compared to bare soils independent of the fertilizer applied and the tillage management. Surprisingly, particularly low abundant Actinobacteria were highly connected in the networks of biocrusts. In contrast, Cyanobacteria were rarely connected, which indicates reduced importance within the microbial community of the biocrusts. However, in bare soil networks, Cyanobacteria were the most connected bacterial group and, hence, might play a role in early biocrust formation due to their ability to, e.g., fix nitrogen and thus induce hotspot-like properties. The microbial community composition differed and network complexity was reduced by conventional tillage. Mineral and organic fertilizers led to networks that are more complex with a higher percentage of positive correlations favoring microbe-microbe interactions. Our study demonstrates that biocrusts represent a microbial hotspot on soil surfaces under agricultural use, which may have important implications for sustainable management of such soils in the future.

## 1. Introduction

Biological soil crusts (biocrusts) are an assemblage of soil particles, photoautotrophic primary producers, heterotrophic microorganisms and microfauna on or within the first few millimeters of the soil surface (Weber et al., 2022). They are particularly abundant in arid and nutrient-poor environments (Belnap, 2003; Maier et al., 2018). In those ecosystems biocrusts fulfill important functions related to carbon and nitrogen fixation, the storage of water and nutrients as well as the induction of soil formation and stabilization (Belnap et al., 2001; Costa et al., 2018). Phototrophic biota such as Cyanobacteria, micro-algae, or mosses are essential key-players to provide such functions (Miralles et al., 2012). For example, Cyanobacteria can stabilize the soil matrix due to their filamentous growth (Chamizo et al., 2018; Jung et al., 2018) and the production of sticky exopolysaccharides (Cania et al., 2019a). The fixation of carbon and nitrogen attracts heterotrophic microbes including Bacteria, Fungi, Archaea and Protists (Baumann et al., 2018; Maier et al., 2018; Roshan et al., 2021). As a consequence, Cyanobacteria are often described as keystone taxa of biocrusts in studies, which investigated microbial community composition and performed correlation network analyses (Chilton et al., 2018; Pombubpa et al., 2020).

There is increasing evidence that biocrust formation is not confined to nutrient-poor and arid regions. Recent studies identified biocrusts in managed ecosystems of mesic regions (Gall et al., 2022) like forests (Baumann et al., 2017; Glaser et al., 2018; Kurth et al., 2021; Glaser et al., 2022a). In contrast to arid biocrusts, they occur here as ephemeral stages. In forests, it was demonstrated that the biomass of biocrusts quickly increased in spring before herbal plant growth or after disturbance events like tree cutting or wind driven tree fall, which gives phototrophic biota of biocrusts a selective advantage (Kurth et al., 2021). Similar to forests, agroecosystems also provide potential niches for biocrust development, such as the time between harvest and sowing or between the rows of broad leave crops like corn, potatoes, or sugar beets. Besides the potential time and space for biocrust development in agroecosystems, multiple studies have reported that single components of agricultural management including tillage (Curaqueo et al., 2010; Wang et al., 2016; Wagg et al., 2018) and fertilization are detrimental for microbial community assembly, which might hamper the development of biocrust communities (Brankatschk et al., 2013; Schulz et al., 2013; Maier et al., 2018). Additionally, the previously described central role of Cyanobacteria in microbial networks might be obsolete in biocrusts of fertilized soils, because of the high external nutrient input. However, ‘on-farm’ studies, which investigate the combined influence of tillage intensity and fertilizer type or amount on microbial community composition, are still missing. Taking the positive effect of biocrusts on many ecosystem functions and their potential importance for sustainable agricultural management into account, there is a strong need to overcome this limitation.

Thus, this study aimed to investigate the combined effect of mineral fertilization and organic treatments as well as tillage intensity on: (1) the composition and correlation of microbial communities in biocrusts of an agricultural field and (2) the ability of those biocrusts to further increase the amount of available nutrients. Samples were taken under the auspices of a long-term fertilization experiment in Germany, which combined different tillage intensities (minimal, reduced, conventional tillage) with different fertilization treatments (different levels of mineral nitrogen fertilizer, with or without crop residue retention). Long-term data revealed already that reduced tillage and crop residue retention increased carbon stocks and sugar beet yields in this experiment (Armbruster et al., 2009, 2012). Sampling was conducted in autumn at the end of sugar beet cultivation but before harvest, which allowed a maximum period for biocrust development. Samples were taken between sugar beet rows. We analyzed nutrient concentrations, the community composition of Archaea, Bacteria and Fungi as well as their co-occurrence and mutual exclusion pattern in biocrusts in comparison to the surrounding bare soil.

## 2. Methods

### 2.1. Experimental field and sampling procedure of biocrusts

**Figure 1:**
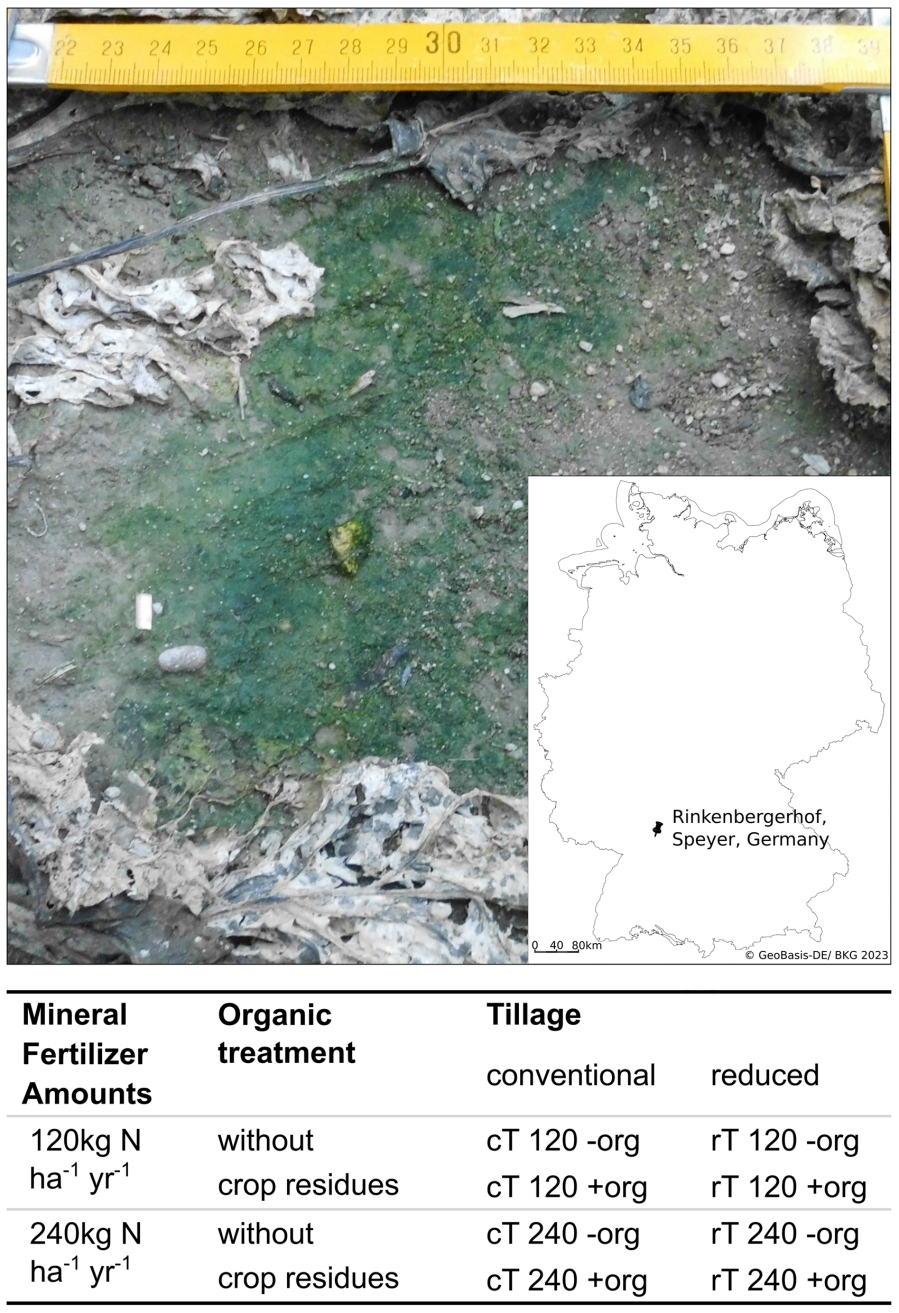
Picture of a biocrusts from a plot with conventional tillage, without crop residues and a mineral fertilization amount of 240  kg  N  ha^−1^ yr.^−1^, taken on October 5th, 2016. Integrated is a map of Germany showing the location of the experimental field. The table below the figure shows the details of treatment combinations for tillage, mineral fertilizer and organic treatment. Tillage management is compared between conventional (cT) and reduced tillage (rT). The effect of mineral fertilizer amounts is evaluated in levels of 120 and 240  kg  N  ha^−1^ yr.^−1^. Organic treatment as crop residues (+org) are compared to a control plot (−org) where crop residues were removed after harvesting.

The current study was conducted 2016 at the agricultural experimental farm “Rinkenbergerhof” in Germany (49°21′34.0”N 8°25′14.8″E), which belongs to the Agricultural Investigation and Research Institute Speyer (Figure 1). Samples were taken in frame of the long-term “International Organic Nitrogen Fertilization Experiment (IOSDV),” which is carried out since 1983. The site is characterized by a mean annual temperature of 10.0°C and a mean annual precipitation of 593 mm. The soil of the IOSDV experiment has been described as Cambisol consisting of equal amounts of silt and sand and 9% of clay. It has a field capacity of 10%. The German arable assessment (“Ackerzahl”) evaluated the soil with 25 to 30, which represents sites with a low soil quality (Bischoff and Emmerling, 1997; Armbruster et al., 2012; Schmid et al., 2018). Phosphorus, potassium and magnesium were applied in equal amounts to all plots (31 kg of phosphorus ha^−1^ yr.^−1^, 121 kg of potassium ha^−1^ yr.^−1^ and 28 kg of magnesium ha^−1^ yr.^−1^). In 2016 during sugar beet cultivation pesticides including fungicides were used based on the particular needs and irrigation of 110 mm was applied to avoid drought damage of the sugar beet plants.

The IOSDV experiment investigates the combination of mineral fertilization and organic treatments (since 1983) as well as tillage intensity (since 2004) in a 3 years crop rotation of sugar beet, winter wheat, and winter barley (Bischoff and Emmerling, 1997; Armbruster et al., 2012; Schmid et al., 2018). The experiment is designed in a full-factorial block design with three replicates per treatment and a plot size per treatment of 6 m * 7.5 m. In frame of the experiment, we sampled the following treatments: (1) 120 and 240 kg N ha^−1^ yr.^−1^ mineral nitrogen fertilization (calcium ammonium nitrate). The applied rates are typical rates used in low-and high input agriculture in the region of the study, respectively. (2) We compared the additional organic treatment (+org) to control plots (−org). This was characterized by the retention of crop residues after harvest and the cultivation of a cover crop (*Raphanus sativus* var. *oleiformisis*) every third year after winter barley cultivation. (3) Tillage intensity was investigated by sampling plots with reduced (rT) and conventional tillage (cT). During rT the soil is broken up by a cultivator to a depth of 10 cm without turning the soil and under cT, the soil is plowed to 30 cm. Because of the different treatments, carbon stocks varied between the treatments.

Sampling took place on October 5 in 2016. The sampling time was chosen to allow for a maximal development of biocrusts and prior to the harvest of sugar beets, which would destroy the biocrusts, because of the heavy machinery used. On some plots, the biocrusts were so prevalent, that reference sampling of bare soil was challenging, which might have been caused by high precipitation of 25 mm in the 2 weeks before sampling. However, during the sampling and right before there was no rain. In total, 24 biocrusts and 24 bare soil samples were taken, which consisted of three replicates of the eight treatment combinations: cT 120 −org, cT 120 +org, cT 240 −org, cT 240 +org, rT 120 −org, rT 120 +org, rT 240 −org, rT 240 +org (see Figure 1). Biocrusts were visually identified as green covered soils according to the biocrust definition of Weber et al. (2022). Biocrusts were sampled from the top millimeters of the mineral soil between the sugar beet rows and were lifted with a spatula and sampled as a coherent layer of approximately 3 mm thickness. Most of the biocrusts were located in tractor traffic lanes and in little grooves (Supplementary Figure S1). Green biofilms on macroscopic organic matter like decomposed leaves were not considered as biocrusts. Samples from biocrust-free areas (no visible phototrophic biomass), referred to as bare soil, were taken from the top 3 millimeters. A composite of five biocrust and bare soil samples per plot was homogenized to reach sufficient amounts of sampling material. All samples were sieved to a particle size of 2 mm. Approximately, 12 g of fresh soil was stored at 4°C for chemical analysis and *ca.* 2 g was shock frozen on dry ice in the field and stored at −80°C for subsequent molecular analyses.

### 2.2. Chemical and physical soil parameters

Soil pH was analyzed as described in DIN ISO 10390 (International Organization for Standardization, 2005) with the electrode SenTix 61 and pH meter (inoLab pH 720 Level 1, Wissenschaftlich-Technische Werkstätten, Weilheim, DE) in 0.01 M CaCl2 extract of 2 g of fresh soil (Stempfhuber et al., 2015). Nitrate, ammonium, dissolved organic nitrogen (DON) and carbon (DOC), were extracted with 0.01 M CaCl2 solution (in a 1:4 ratio) from 3 g of fresh soil by 45 min overhead shaking at 67 rpm (Reax 2, Heidolph Instruments, Schwabach, Germany), followed by filtration through Whatman™ 595 1/2 filters (Sigma-Aldrich, St. Louis, MO, United States) and determined by a segmented flow analyzer (Skalar SANPlus 5100 with autosampler 1050, Skalar analytic, DE, EU). DOC and DON were analyzed on DIMATOC2000 (DIMATEC Analysentechnik, Essen, DE). To calculate dissolved inorganic carbon (DIC), one drop of 32% HCl was added to the filtrate prior to analysis and the difference to DOC without HCl was used to calculate DIC (Brankatschk et al., 2011). Chlorophyll *a* was used as a proxy for phototrophic biomass and determined according to Ritchie (2008). The procedure was as follows, 0.7 g frozen soil was extracted in 3 mL 96% aqueous ethanol (v/v) and incubated for 30 min at 78°C. Afterwards, the extract was centrifuged at 5°C at 6,000 rpm. The absorption of the supernatant was measured at wavelengths of 632 nm, 649 nm, 665 nm, 696 nm using the spectrophotometer UV-2401PC (Shimadzu, Kyoto, JPN). Extraction was repeated until no chlorophyll could be detected in the supernatant anymore and the content was summed up for all extraction steps.

### 2.3. DNA extraction

DNA was extracted from 0.3 g of soil using the NucleoSpin^®^ Soil kit (Macherey-Nagel, Düren, DE) according to the manufacturer’s manual using lysis buffer SL2 and 150 μL of enhancer. DNA quality was assessed using a UV-VIS spectrophotometer (NanoDrop^®^ ND-1000 spectrophotometer, Thermo Fisher Scientific, Waltham, Massachusetts, United States). DNA concentration was determined using the Quant-IT^™^ Picogreen^®^ dsDNA Assay Kit (Thermo Fischer Scientific). A negative extraction control sample without soil was processed as negative control to check for possible contaminations of chemicals from the kit used for nucleic acid extraction.

### 2.4. Quantification of prokaryotic and fungal biomass

Real-time qPCR was used to determine the abundance of Bacteria, Archaea and Fungi, which was used as a proxy for their biomass. For Bacteria (Bach et al., 2002) and Archaea (Bano et al., 2003; Nicol et al., 2003), kingdom specific primers targeting the V5-V6 (Bacteria) and V2-V5 (Archaea) region of the 16S rRNA gene were used to estimate their absolute abundance separately. For Fungi, primers targeting the ITS1 & 2 region were used (White et al., 1990). SYBR Green^®^ based assays (Applied Biosystems, Foster City, CA, United States) were performed on a 7,300 Real-Time PCR System (Applied Biosystems). Details about forward (F) and reverse (R) primer, reaction conditions, and calibration standards are summarized in Supplementary Table S1. Primers were purchased from Metabion (Planegg, Germany) and bovine serum albumin (BSA) from Sigma-Aldrich (Missouri, United States). To exclude inhibitory effects, dilution tests were performed. Standard series (10^6^ to 10^2^ gene copies μl^−1^) and samples diluted to 1/32 were included in each run. To check for possible contamination of chemicals used, a negative control without DNA of samples was included, as well. To evaluate the quality of the qPCR, melting curve analyses were performed and randomly chosen samples were checked by electrophoresis on a 1.5% agarose gel. The amplification efficiency was calculated by ε = 10^(−1/slope)^−1 with the slope of the standard series, and was 78–84% for all genes. The *r*^2^ of the standard curve was >0.987.

### 2.5. Diversity of prokaryotes and Fungi

As no specific primers exist, which separately target Bacteria and Archaea for Illumina MiSeq^®^ sequencing (Illumina, San Diego, CA, United States), we used the universal primers Arch0519 F (Klindworth et al., 2013) and Pro 805 R (Herlemann et al., 2011), which were optimized for the simultaneous sequencing of both prokaryotes, Bacteria and Archaea, and target the V4 region of the 16S rRNA gene. For Fungi, the ITS 3 primer mix and ITS 4 primer mix (Tedersoo et al., 2015) were used. Primer sequences are given in Supplementary Table S2. Each assay was set to 25 μL and consisted of NebNext^®^ High-Fidelity 2X PCR Master Mix (New England Biolabs, Ipswich, MA, United States), forward (F) and reverse (R) primer with Illumina overhang (Metabion), 3% BSA (Sigma-Aldrich), DEPC treated water, and 1 μL of DNA (3 ng μl^−1^). Primers were purchased from Metabion (Planegg, Germany) and bovine serum albumin (BSA) from Sigma-Aldrich (St. Louis, Missouri, United States). PCR conditions are given in Supplementary Table S2. All samples, PCR negative controls and extraction blanks were amplified in triplicates and checked on 1% agarose gel before pooling. PCR clean-up was carried out with Agencourt AMPure XP magnetic beads (Beckman Coulter Life Sciences, Brea, CA, United States) according to the manufacturer’s protocol with a DNA to bead ratio of 0.8. A quality check to assess DNA concentration and fragment size was performed on Fragment Analyzer™ Automated CE System (Agilent Technologies, Santa Clara, CA, United States). For multiplexing, Nextera^®^ XT Index Kit v2 (Illumina) was used. Each indexing PCR reaction of 25 μL consisted of 12.5 μL NebNext^®^ High-Fidelity 2X PCR Master Mix (New England Biolabs), 2.5 μL of indexed forward and reverse primer (10 pmol/μl) and 10 ng of purified amplicon. The following PCR conditions were used: 30 s at 98°C, followed by 8 cycles of 10 s at 98°C, 30 s at 55°C and 30 s at 72°C and 5 min final elongation at 72°C. PCR clean-up was performed with AMPure Beads (Beckman Coulter Life Sciences) as described before. Both DNA concentration and quality were assessed on the Fragment analyzer (Agilent Technologies). Samples were diluted to 4 nM and 5 μL of each library were pooled for sequencing on the MiSeq^®^ instrument with 2 × 300 bp (Illumina) using MiSeq Reagent kit v3 (600 cycles) and spiked with 20% PhiX as a control. Samples with less than 10,000 reads were re-sequenced.

Sequencing adapters were removed using AdapterRemoval (Schubert et al., 2016). DADA2 package Version 1.8.0 (Callahan et al., 2016) in R Version 3.6.1 (R Core Team, 2022) was used for length and quality filtering including PhiX and chimera removal. For prokaroytes, forward and reverse reads were trimmed at 10 and 250 bp and 10 and 200 bp, respectively. For Fungi, the parameters were set to 10 and 275 bp and 10 and 225 bp, respectively. Alignment of mate pairs, inferring into amplicon sequencing variants (ASVs) and merging of sequencing runs was done prior to taxonomy assignment. For prokaroytes, this was done against the Silva database Version 132 (Quast et al., 2013) and for Fungi, against the Unite database Version 7.1 [2016-11-20, Kõljalg et al. (2013)]. After taxonomy assignment, reads present in samples of blank extraction or PCR negative controls were removed (11 of 9,637 ASVs for prokaroytes, 6 of 3,715 ASVs for Fungi) as well as ASVs assigned as Chloroplasts or Mitochondria (on average 11.99%). To differentiate between Chloroplast sequences (representative chloroplast reads from plants, Algae and Bacteria) and cyanobacterial ASVs detected in our study, a phylogenetic tree was calculated and only sequences clearly assigned as cyanobacteria were processed (Supplementary Figure S2). Random subsampling was performed to the lowest number of reads (prokaroytes: 19,722 reads, Fungi: 22,731 reads) using the function rarefy_even_depth() of the R package phyloseq Version 1.30.0 (McMurdie and Holmes, 2013) with the random seed set to 3,006. As they were no longer present after subsampling, 448 ASVs out of 9,626 ASVs for prokaroytes and 283 ASVs out of 3,709 ASVs for Fungi have been removed.

The sequence data was submitted to NCBI via the Sequence Read Archive (SRA) and is available under the accession number PRJNA646655.

### 2.6. Statistical data analysis

Data analysis was performed with R Version 3.6.1 (R Core Team, 2022). The vegan package Version 2.5-7 was used to calculate rarefaction curves, alpha diversity [Shannon diversity (S) (Shannon, 1948), richness as number of ASVs (R) and Pielou’s eveness (P) (Pielou, 1966)] of the subsampled data. To test for the normal distribution of the data residual vs. fitted plots and sample quantile vs. theoretical quantile plots were tested for normal distribution and homogenous variance to verify the models. If needed, data was transformed to meet normal distribution with the 1 + log transformation for abiotic parameters, gene abundances, and alpha diversity; square root transformation for taxa on family level with abundances of more than 2% for at least one replicate of Bacteria and 3% of Fungi. Linear models were applied to detect significant differences according to biocrust presence or treatment for soil parameters, absolute abundances of microbial groups, alpha diversity (S, R, P) and relative abundance of microbial families obtained from sequencing results. To disentangle treatment effects (tillage, mineral fertilization amount, and organic treatment), biocrust and bare soil samples were separated.

Difference in β-diversity of the treatments was calculated by Bray-Curtis-distance, plotted as nonmetric multidimensional scaling (NMDS) plots (Oksanen et al., 2017) and tested for significance by PERMANOVA. The barplots and heatmaps were created using ggplot2 package Version 3.3.5 (Wickham, 2009). CoNet (Faust and Raes, 2016) as an add-on of Cytoscape Version 3.7.2 (Shannon et al., 2003) was used to calculate correlation networks on relative abundance data. For network calculations, ASVs unclassified at the phylum, class and order level were removed. Relative abundance was calculated separately for prokaryotes and Fungi and data sets were merged afterwards. Networks were calculated separately for the two sample (biocrust and bare soil) and management types (tillage: cT and rT, mineral fertilizer: 120 and 240, organic treatment: -org and + org) to detect differences in co-occurrence patterns (*N* = 12). To obtain correlations only valid for all treatments, ASVs present in less than 10 samples were excluded from analysis. Significant correlations (co-occurrences vs. mutual exclusion) were determined based on Pearson and Spearman correlation as well as Bray-Curtis and Kulback-Leibler dissimilarity. At least two of the analyses need to support the link connecting two nodes. 1,000 permutations and bootstrap scores were generated with Brown value of p merging (Brown, 1975). Gephi 0.9.2 (Bastian et al., 2009) was used for visualization of undirected networks with the Fruchterman-Reingold layout (Fruchterman and Reingold, 1991). Correlation partners are visualized with the chordDiagram function of circlize package Version 0.4.13 in R (Gu et al., 2014). Network hubs were defined as highest connected nodes (Tipton et al., 2018), where hubs not specified at the family level and lower were ignored.

## 3. Results

### 3.1. Basic properties of biocrusts

**Figure 2:**
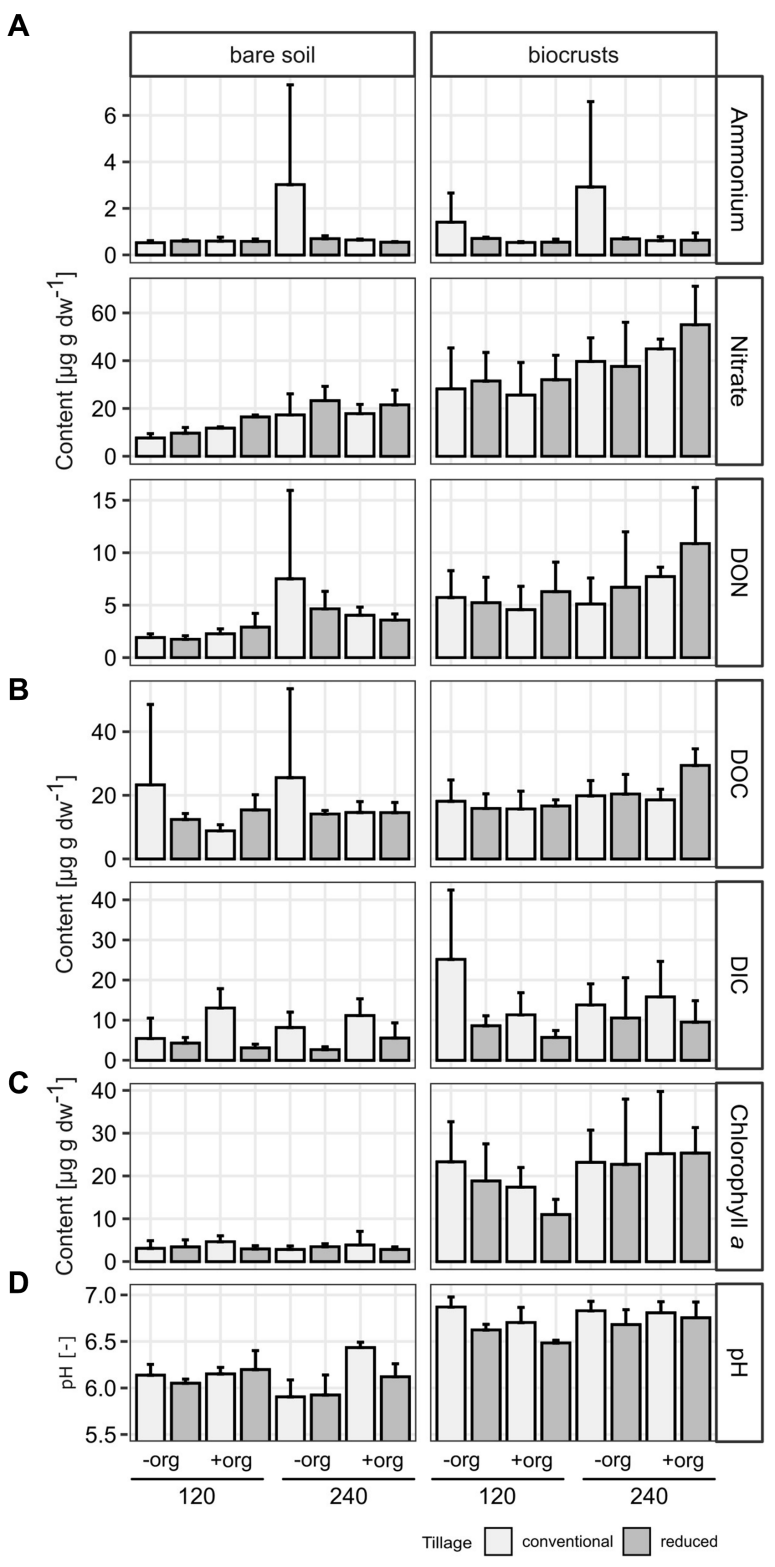
Soil properties of **(A)** nitrogen [ammonium, nitrate, and dissolved organic nitrogen (DON)] and **(B)** carbon [dissolved organic (DOC) and inorganic (DIC) carbon] pools, **(C)** chlorophyll a [μg per g soil dry weight (dw)], and **(D)** pH are given as mean values as the average of replicates (*n* = 3) in bar plots with error bars given as standard deviation. Treatments are given for tillage [conventional (cT) vs. reduced tillage (rT)], organic treatment [without (−org) vs. with crop residues (+org)], and mineral fertilization amount (120 vs. 240 kg N/ha·a).

**Figure 3:**
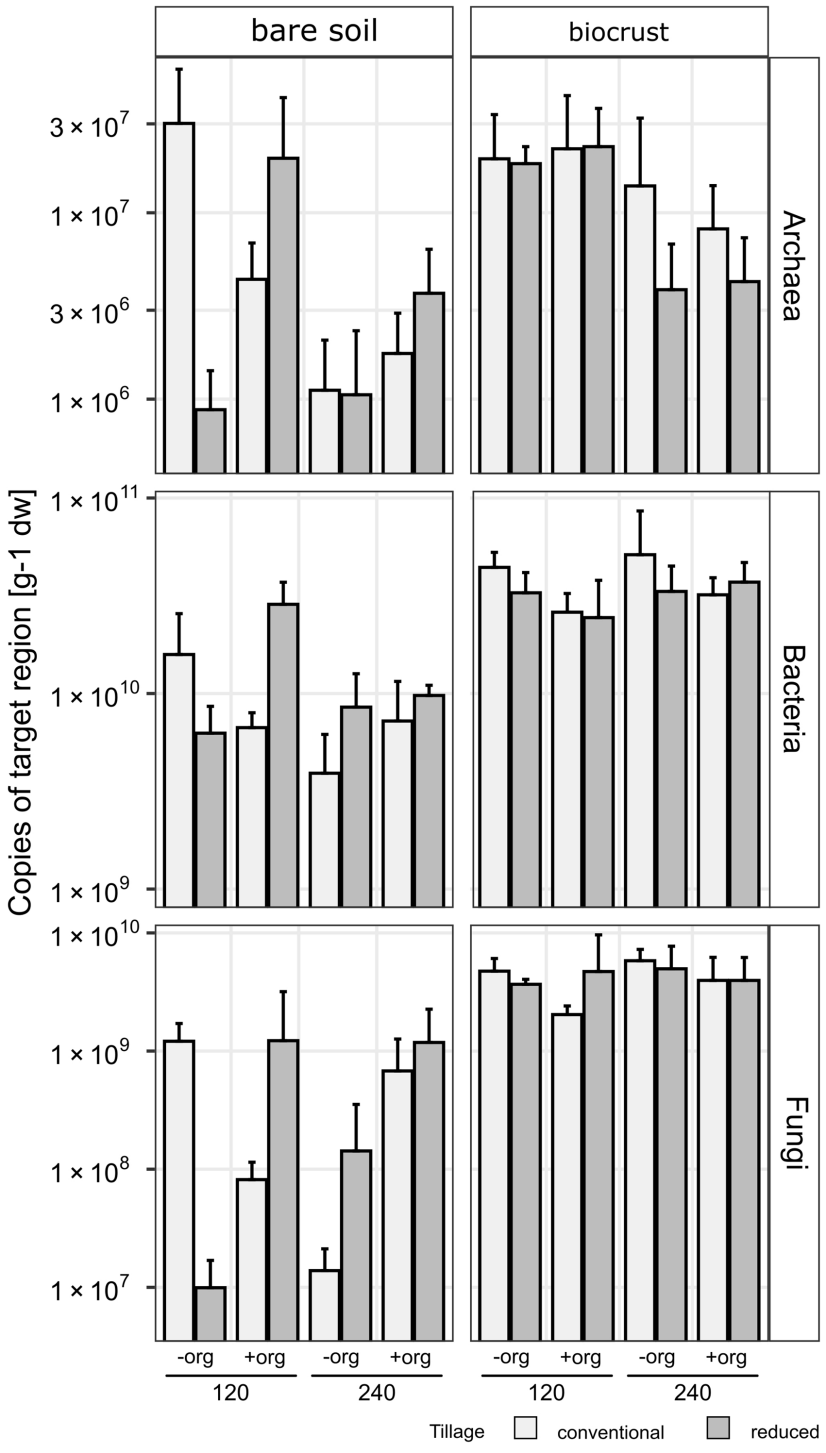
Copies of target region [per gram soil dry weight (dw)] 16S rRNA gene for Archaea and Bacteria and ITS region for Fungi in bar plots as the average of replicates (*n* = 3) and error bars displaying the standard deviation. Treatments are given for tillage [conventional (cT) vs. reduced tillage (rT)], organic treatment [without (−org) vs. with crop residues (+org)] and mineral fertilization amount (120 vs. 240  kg N/ha·a).

The content of phototrophic biomass measured as chlorophyll *a* was 6-times higher in biocrusts compared to bare soil (*F* = 183.7, *p* < 0.001) reaching 17.87 ± 7.2 μg g^−1^ soil dry weight (dw) in biocrusts compared to 2.98 ± 1.2 μg g^−1^ dw in bare soil (Figure 2; Table 1; Supplementary Table S3). The management had no significant influence on the chlorophyll *a* content. The microbial biomass, determined as the amount of extracted DNA (*F* = 18.5, *p* < 0.001) and the abundance of Bacteria, Archaea and Fungi was significantly higher (18.7 ≥ *F* ≤ 106.8, *p* < 0.001) in biocrusts compared to bare soils (Supplementary Table S4). Bacterial 16S rRNA genes (2.3*10^10^ ± 8.4*10^9^ copies g^−1^ dw) were significantly (*F* = 277, *p* < 0.001) more abundant, than Fungal ITS sequences (2.4*10^9^ ± 1.3*10^9^ copies g^−1^ dw) and archaeal 16S rRNA genes (1.1*10^7^ ± 8.9*10^6^ copies g^−1^ dw) (Figure 3; Table 1; Supplementary Table S5). In biocrusts, the bacterial and fungal abundance was not influenced by management. Contrastingly in bare soils, tillage, mineral fertilizer amount and organic treatments altered archaeal, bacterial and fungal biomass. For example, in bare soils fungal biomass was significantly higher with organic treatments (*F* = 6.7, *p* = 0.02). Further, an interacting treatment effect for mineral fertilizer and organic treatments were observed and in samples with 120 kg N ha^−1^ yr.^−1^ the fungal abundance was significantly reduced with organic treatment (*F* = 6.1, *p* = 0.025). Although, a similar pattern was observed for bacterial abundance this was not significant (*F* = 3.6, *p* = 0.059). Archaeal biomass was reduced in biocrusts and bare soils in the treatment with mineral fertilizer of 240 kg N ha^−1^ yr.^−1^ (*F* > 10.8, *p* ≤ 0.05).

**Table 1:** *F* and *p* values of linear models for the effect of biocrust presence, as well as separated for both compartments (bare soil, biocrust) the effect of tillage (cT vs. rT), mineral fertilizer amount (120 vs. 240) and organic treatment (−org vs. +org) as well as their interacting effects on 1 + log transformed values of soil properties [ammonium, nitrate and dissolved organic nitrogen (DON), dissolved organic (DOC) and inorganic (DIC) carbon], chlorophyll *a*, pH and abundances of Archaea, Bacteria, and Fungi determined by qPCR.

Variable	Biocrust effect		Compartment	Tillage		Mineral fertilizer amount		Organic treatment		Tillage * organic treatment		Tillage * mineral fertilizer amount		Mineral fertilizer amount * organic treatment		Tillage * mineral fertilizer amount * organic treatment		*F* value	*p* value		*F* value	*p* value	*F* value	*p* value	*F* value	*p* value	*F* value	*p* value	*F* value	*p* value	*F* value	*p* value	*F* value	*p* value
Ammonium	0.5	0.475	Bare soil	0.54	0.472	1.48	0.241	0.88	0.363	0.14	0.714	1.07	0.317	1.27	0.276	0.45	0.514
			Biocrust	1.98	0.178	0.39	0.541	5.03	**0.039**	1.74	0.205	0.23	0.637	0.04	0.845	0.18	0.679
Nitrate	57.5	**<0.001**	Bare soil	6.25	**0.024**	27.18	**0.000**	5.44	**0.033**	0.02	0.884	0.01	0.931	5.11	**0.038**	0.39	0.541
			Biocrust	0.60	0.450	5.74	**0.029**	0.52	0.483	0.26	0.620	0.43	0.520	0.67	0.425	0.08	0.780
DON	17.2	**<0.001**	Bare soil	0.03	0.874	12.98	**0.002**	0.05	0.832	0.11	0.749	0.17	0.688	1.85	0.192	0.13	0.723
			Biocrust	0.28	0.602	1.08	0.313	1.42	0.250	0.50	0.490	0.00	0.993	1.75	0.205	0.03	0.869
DOC	5.5	**0.025**	Bare soil	0.02	0.904	0.54	0.472	0.37	0.550	1.12	0.305	0.28	0.606	0.07	0.801	0.41	0.529
			Biocrust	1.05	0.320	6.32	**0.023**	0.33	0.574	2.20	0.157	1.25	0.281	0.81	0.382	0.27	0.608
DIC	10.1	**0.003**	Bare soil	12.99	**0.002**	0.35	0.565	3.98	0.063	1.82	0.196	0.32	0.579	0.01	0.914	3.79	0.069
			Biocrust	5.50	**0.032**	0.02	0.879	0.19	0.667	0.35	0.562	0.01	0.912	1.88	0.190	0.01	0.938
Chlorophyll *α*	183.7	**<0.001**	Bare soil	0.11	0.746	0.19	0.665	0.22	0.645	1.55	0.230	0.39	0.540	0.48	0.498	0.08	0.778
			Biocrust	0.90	0.357	2.55	0.130	0.42	0.528	0.00	0.965	0.81	0.380	1.59	0.225	0.39	0.539
pH	236.9	**<0.001**	Bare soil	1.89	0.188	0.50	0.488	13.80	**0.002**	0.67	0.425	1.05	0.322	5.71	**0.029**	3.66	0.074
			Biocrust	11.25	**0.004**	3.85	0.067	1.67	0.214	0.35	0.562	1.75	0.205	3.21	0.092	0.14	0.716
Archaea	18.7	**<0.001**	Bare soil	0.54	0.472	10.83	**0.005**	3.67	0.073	6.40	**0.022**	1.97	0.180	0.46	0.506	4.52	**0.049**
			Biocrust	0.33	0.571	11.71	**0.003**	0.06	0.817	0.00	0.990	0.94	0.347	0.00	0.976	0.00	0.998
Bacteria	99.1	**<0.001**	Bare soil	4.95	**0.041**	6.25	**0.024**	3.55	0.078	4.15	0.059	0.62	0.444	0.00	0.983	8.89	**0.009**
			Biocrust	0.91	0.356	1.18	0.293	2.92	0.107	0.92	0.351	0.20	0.658	1.23	0.283	0.20	0.662
Fungi	106.8	**<0.001**	Bare soil	1.01	0.329	0.26	0.616	6.65	**0.020**	3.35	0.086	6.66	**0.020**	6.08	**0.025**	6.48	**0.022**
			Biocrust	0.00	0.983	1.23	0.284	3.06	0.099	1.19	0.292	0.28	0.604	0.11	0.748	0.17	0.685

Significant values (*p* < 0.05) are marked in bold.

The development of biocrusts came along with changes in abiotic soil properties. In general, water content, (*F* = 7.78, *p*  < 0.001; biocrusts 13.7% ± 2.8, bare soils 11.6% ± 2.1), pH (6.71 ± 0.2), nitrate (36.79 ± 12.7 μg g^−1^ dw), DON (6.52 ± 3.0 μg g^−1^ dw), DOC (19.3 ± 4.8 μg g^−1^ dw) and DIC (12.53 ± 7.1 μg g^−1^ dw) were up to 2.4 times higher (236.9 > *F* > 5.5, *p* < 0.03) in biocrusts compared to bare soils (Figure 2; Table 1; Supplementary Table S3). Only ammonium values were similar (*p* = 0.475) in biocrusts (1.004 ± 0.7 μg g^−1^ dw) and bare soils (0.898 ± 0.6 μg g^−1^ dw) but revealed very high standard deviations. In biocrusts, cT further increased pH (6.80) and DIC (16.51 μg g^−1^ dw) compared to rT (6.34 and 8.56 μg g^−1^ dw), while higher mineral N fertilization increased nitrate (29.28–44.30 μg g^−1^ dw) and DOC (16.57–22.03 μg g^−1^ dw) concentrations significantly (*F* > 5.5, *p* < 0.003). In bare soils, pH increased due to organic treatments (6.00–6.23), while nitrate (11.33–20.24 μg g^−1^ dw) and DON (2.20–6.08 μg g^−1^ dw) increased significantly (*F* > 13, *p* < 0.002) due to higher mineral fertilization. Only in bare soils, the interaction of mineral fertilization amount and organic treatments had significant effects (*F* > 5.1, *p* < 0.04) on nitrate and pH (Figure 2; Table 1).

### 3.2. Microbial diversity in biocrusts

In total, 3,601,616 reads were obtained from 16S rRNA gene amplicon sequencing and 4,995,629 reads from ITS amplicon sequencing. The average loss of reads because of the different filter steps during data processing was 18.9% for prokaryotes and 29.4% for Fungi (Supplementary Tables S6, S7). However, after subsampling, all rarefaction curves still reached a plateau indicating a sufficient sampling depth for further analysis (Supplementary Figure S3). Details of all sequencing runs including number of demultiplexed, filtered and merged reads are summarized in the Supplementary Tables S6, S7.

For α-diversity indices of prokaryotes, no difference between bare soils and biocrusts was detected (*F* < 0.6, *p* > 0.4). In contrast, fungal diversity and richness were significantly (*F* > 8.8, *p* < 0.005) lower in biocrusts (*S* = 3.97 ± 0.35, *R* = 274 ± 88) compared to bare soils (*S* = 4.35 ± 0.43, *R* = 390 ± 110), while evenness was also not affected. Regarding the management, the evenness of prokaryotes was reduced in cT compared to rT in both compartments (*F* > 6, *p* < 0.03) (Supplementary Tables S8, S9), while tillage had contrasting effects on fungal α-diversity in bare soils and biocrusts. For example, rT compared to cT caused a decrease in fungal richness in bare soils (*F* = 11.9, *p* = 0.003) and an increase in biocrusts (*F* > 6.6, *p* < 0.02). Interacting treatment effects (*p* < 0.05) were detected in biocrusts for archaeal/bacterial richness (Tillage * Mineral Fertililzer Amount * Organic Treatment) and fungal diversity and richness (Tillage * Organic Treatment) (Supplementary Tables S8, S9).

Beta-diversity analysis revealed significant differences between biocrust and bare soil microbial communities (*p* < 0.02) as well as between the different treatments (*p* < 0.05) (Table 2; Supplementary Figures S4, S5). Regarding the prokaryotic community, the presence of biocrusts reduced treatment effects on the community composition. The fungal community composition was affected by all treatments no matter if biocrusts or bare soils were considered (*p* < 0.05).

**Table 2:** Anova Results on Bray-Curtis-Distance-Matrix of Community data on ASV Level.

**Kingdom**	**Compartment**	**Management effect**		
			**r** ^**2**^	**p**
Prokaryotes	Biocrust vs. Bare soil		0.060	0.001
	Biocrust	Tillage	0.123	**0.001**
		Mineral Fertilizer Amounts	0.084	**0.005**
		Organic Treatment	0.043	0.236
		Tillage * Mineral Fertilizer Amounts	0.047	0.138
		Tillage * Organic Treatment	0.047	0.143
		Mineral Fertilizer Amounts * Organic Treatment	0.033	0.542
		Tillage * Mineral Fertilizer Amounts * Organic Treatment	0.046	0.149
	Bare soil	Tillage	0.092	**0.001**
		Mineral Fertilizer Amounts	0.082	**0.007**
		Organic Treatment	0.087	**0.002**
		Tillage * Mineral Fertilizer Amounts	0.032	0.591
		Tillage * Organic Treatment	0.054	0.077
		Mineral Fertilizer Amounts * Organic Treatment	0.030	0.710
		Tillage * Mineral Fertilizer Amounts * Organic Treatment	0.031	0.701
Fungi	Biocrust vs. Bare soil		0.040	0.013
	Biocrust	Tillage	0.155	**0.001**
		Mineral Fertilizer Amounts	0.091	**0.006**
		Organic Treatment	0.097	**0.002**
		Tillage * Mineral Fertilizer Amounts	0.061	**0.049**
		Tillage * Organic Treatment	0.053	0.087
		Mineral Fertilizer Amounts * Organic Treatment	0.025	0.616
		Tillage * Mineral Fertilizer Amounts * Organic Treatment	0.016	0.921
	Bare soil	Tillage	0.100	**0.004**
		Mineral Fertilizer Amounts	0.082	**0.007**
		Organic Treatment	0.101	**0.002**
		Tillage * Mineral Fertilizer Amounts	0.015	0.989
		Tillage * Organic Treatment	0.064	**0.033**
		Mineral Fertilizer Amounts * Organic Treatment	0.033	0.532
		Tillage * Mineral Fertilizer Amounts * Organic Treatment	0.025	0.803

Significant values (*p* < 0.05) are marked in bold.

### 3.3. Microbial community composition in biocrusts

**Figure 4:**
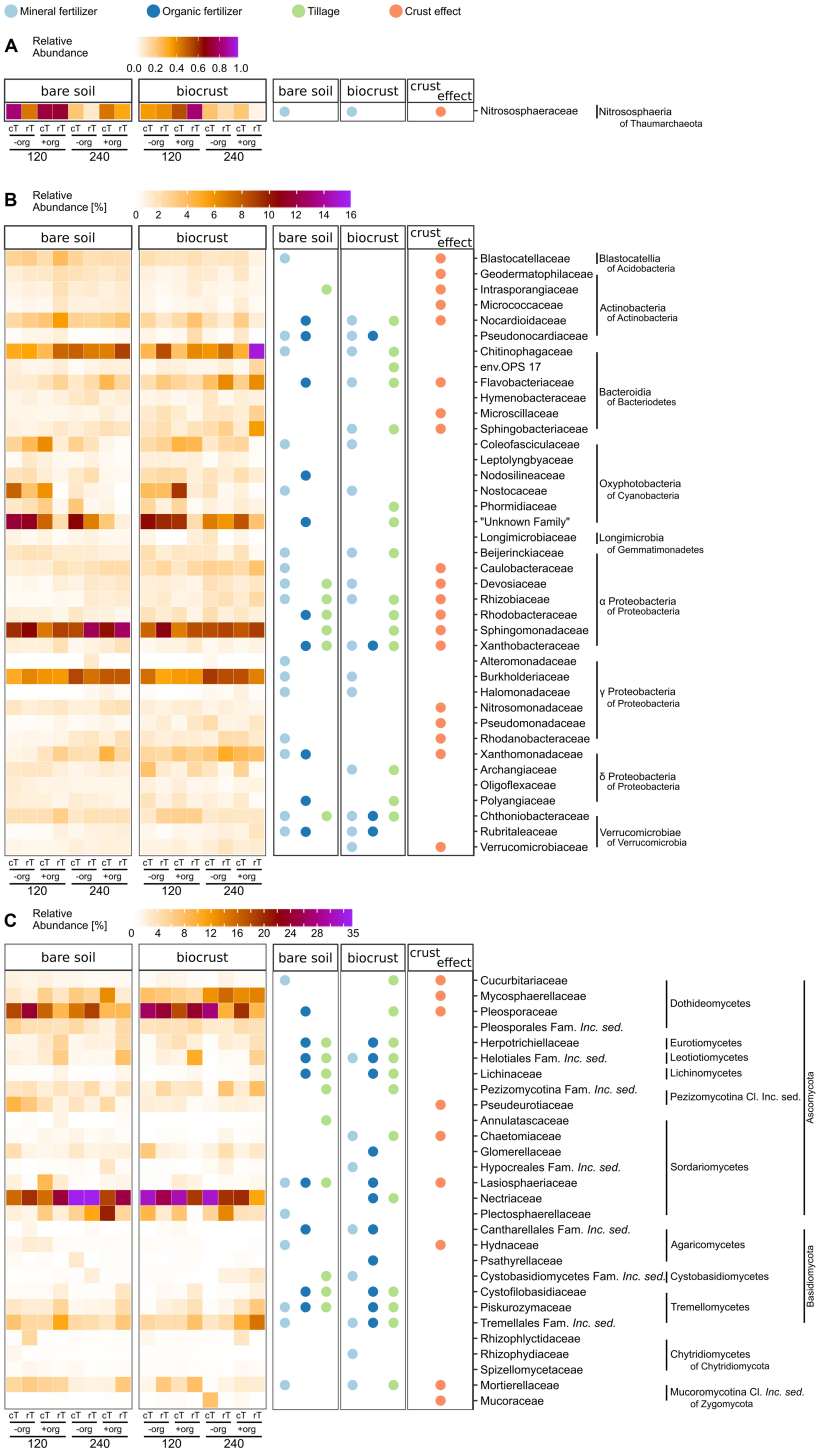
Heat maps of **(A)** archaeal, **(B)** bacterial, and **(C)** fungal community composition in bare soils and biocrusts at the family level are displayed in the left two columns (for Bacteria **(B)** and Fungi **(C)** with an abundance above 2% in at least one replicate). The average of field replicates (*n* = 3) is presented. Values range from white (0%), orange, and dark red to the highest abundance in purple **(A)** 1%, **(B)** 16%, **(C)** 35%). Significant variations (*p* < 0.05) are shown in the right columns for the biocrust effect (bare soil vs. biocrust) (orange), mineral fertilizer amount (light blue), organic treatment (dark blue), and tillage (green) separately for bare soils and biocrusts. The families additionally influenced by interacting treatment effects are supplied in Supplementary Tables S10, S11.

In total, 2,860 different bacterial and 48 archaeal families were detected, of which 39 bacterial families were highly abundant (at least 2% abundance in one of the samples) (Figure 4B). Predominant families were *Sphingomonadaceae* (bare soils 10.1 ± 1.9%, biocrusts 8.6 ± 1.2%), *Burkholderiaceae* (bare soils 7.0 ± 1.4%, biocrusts 7.2 ± 1.5%), “Unknown Family” of Oxyphotobacteria (bare soils 6.8 ± 4.3%, biocrusts 7.1 ± 3.1%), *Chitinophagaceae* (bare soils 3.6 ± 1.6%, biocrusts 7.1 ± 3.8%), and *Flavobacteriaceae* (bare soils 2.1 ± 1.0%, biocrusts 3.6 ± 1.8%). The most abundant archaeal family was *Nitrososphaeraceae* with 0.34 ± 0.30% of abundance in biocrusts and 0.48 ± 0.3% in bare soils (Figure 4A).

Overall, 19 bacterial families were significantly (*p* < 0.03) different between biocrusts and bare soils (Figure 4B). Eleven of those were higher in relative abundance in biocrust samples, including many reads which were assigned as α-and γ-Proteobacteria, while reads corresponding to Acidobacteria and Actinobacteria were lower abundant in biocrusts compared to bare soils. Interestingly, the relative abundance of ASVs linked to cyanobacterial families was similar in biocrusts (16.8 ± 9.0%) and bare soils (16.0 ± 13.3%).

The amount of mineral fertilizer addition significantly (4.6 < *F* < 23.0, *p* < 0.05) changed the relative abundance of 54% of the highly abundant bacterial families in biocrusts and bare soils. The identity of the affected families was mostly the same in biocrusts and bare soils and included the highly abundant *Burkholderiaceae* and *Chitinophagaceae.* Regarding Cyanobacteria, the relative abundance of *Coleofasciculaceae* and *Nostocaceae* was seven times higher in treatments with 120 kg N ha^−1^ y^−1^ compared to 240 kg N ha^−1^ y^−1^ in both biocrusts and bare soils (*F* > 5.8, *p* < 0.02). In contrast, the effect of crop residues was less pronounced, especially in biocrusts only 10% (compared to 26% in bare soils) of the dominant bacterial families were positively affected and had higher abundances due to the organic treatment. Of those, *Pseudonocardiaceae*, *Xanthobacteraceae*, *Chthoniobacteraceae*, and *Rubritaleaceae* were affected by mineral fertilizer and organic treatmentor the interaction of both (*Pseudonocardiaceae*, *Chthoniobacteraceae*) (Figure 4B).

Tillage significantly (15.1 < *F* < 65.3, *p* < 0.04) changed the relative abundance of 15 bacterial families in biocrusts, but only seven in bare soils. For example, in biocrusts *Chitinophagaceae* were increased by cT (4.7 ± 1.4% to 9.4 ± 4.3%) (*F* ≥ 23.0, *p* < 0.001) while the “Unknown Family” of Oxyphotobacteria (including also species of *Leptolyngbya* in Silva database v132) decreased under rT (8.8 ± 3.3% to 5.3 ± 3.7%) (*F* ≥ 8.0, *p* ≤ 0.01). Interacting treatment effects were mainly observed, where one of the factors alone significantly influenced the abundance of bacterial families (Supplementary Table S10). Only the family *env. OPS_17* (Bacteroida of Bacteroidetes) in biocrusts was significantly (*F* = 6.4, *p* = 0.027) affected by all treatments in combination but not by one single one. *Longimicrobiaceae* in biocrusts were significantly (*F* = 6.0, *p* = 0.027) affected by tillage and in combination with mineral fertilization amount without these two affecting the family individually. In general, more interacting effects were observed in biocrusts (*n* = 11) than in bare soils (*n* = 5).

Two hundred and twenty different fungal families were detected. In our analyses we focused on the 29 most abundant ones (at least 2% abundance in one of the samples) (Figure 4C). Predominant families were *Nectriaceae* (bare soils 22.6 ± 6.9%, biocrusts 23.1 ± 7.2%), *Pleosporaceae* (bare soils 14.4 ± 5.6%, biocrusts 19.4 ± 7.1%), *Mycosphaerellaceae* (bare soils 4.9 ± 3.6%, biocrusts 9.8 ± 4.1%), Tremellales Fam. *Incerta sedis* (*Inc. sed.*) (bare soils 5.6 ± 2.4%, biocrusts 6.4 ± 3.9%), and *Plectosphaerellaceae* (bare soils 6.5 ± 6.4%, biocrusts 5.3 ± 4.1%). One third of the fungal families significantly (4.31 < *F* < 12.1, *p* < 0.05) differed in relative abundance between biocrusts and bare soils, of which many families were 1.3–4.5 times more abundant in bare soils (Figure 4C). The fungal community in biocrusts was more sensitive toward all management practices than bare soil communities. For example, 44% of the dominant families in biocrusts were significantly influenced by cT, of which the very high abundant families *Nectriaceae* (cT: 27.7%, rT: 18.5%) (*F* = 9.1, *p* = 0.008) and *Pleosporaceae* (cT: 22.4%, rT: 16.3%) (*F* = 5.1, *p* = 0.04) were significantly increased while the others were lower compared to rT. Additionally, +org influenced more fungal families in the biocrusts (39%) compared to bare soils (29%). However, *Herpotrichiellaceae*, Helotiales Family *Inc. sed.*, *Lasiosphaeriaceae*, *Lichinaceae*, Cantharellales Family *Inc. sed.*, *Cystofilobasidiaceae* (in bare soil not significantly), and *Piskurozymaceae* were increased up to six times in both, bare soils (*F* > 5.5, *p* < 0.02) and biocrusts (*F* > 4.6, *p* < 0.05) under +org. Mineral fertilizer alone changed the abundance of the same number of families in biocrusts and bare soils, but their identity mostly differed. Interacting treatment effects were equally distributed in bare soils and biocrusts (*n* = 9) (Supplementary Table S11). In bare soils, Pleosporales Fam. *Inc. sed.*, Tremellales Fam. *Inc. sed.* and *Mucoraceae* were affected by tillage combined with organic treatment, and for Cystobasidiomycetes Fam. *Inc. sed.* of mineral fertilizer and organic treatment combined was observed, without the single effects influencing those fungal families (Supplementary Table S11).

### 3.4. Cross-kingdom correlation networks in biocrusts

**Figure 5:**
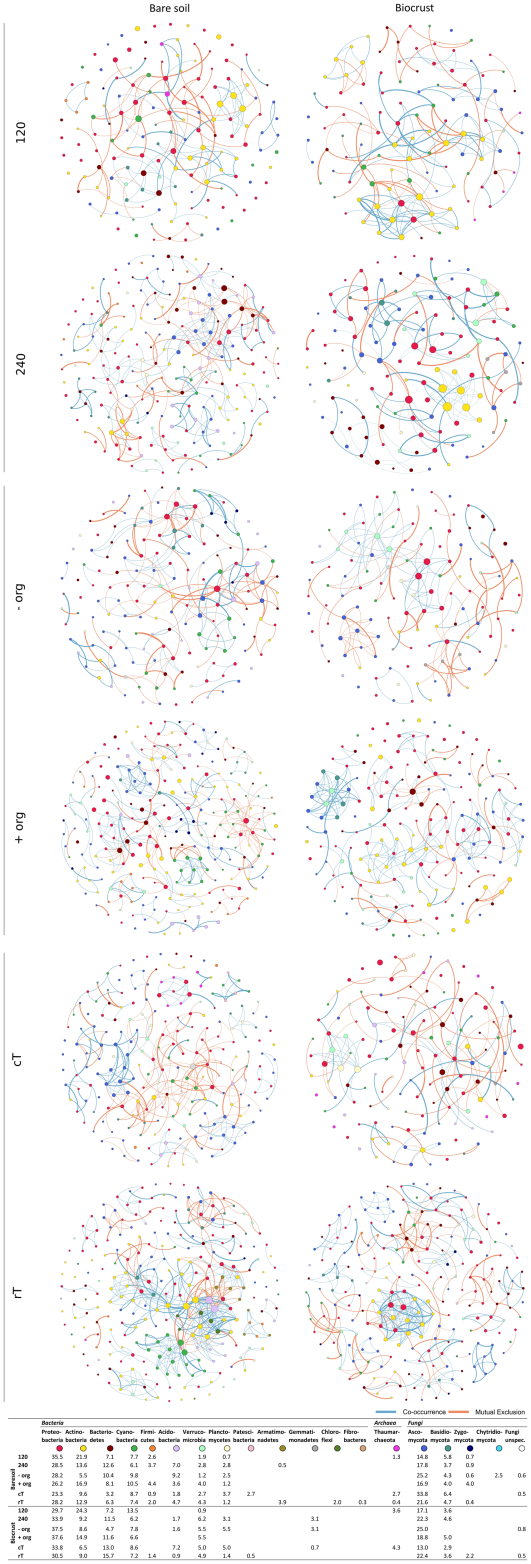
Networks in Fruchterman-Reingold layout based on significant ASV correlation analysis for bare soil and biocrust for each management variation (*n* = 12). Nodes are colored by phylum and the color code is given below the network figures, where also the abundance of phyla within the networks is given. Red connections display negative and green connections display positive correlations. The number of correlations defines the size of one node. The lower the *p* value of the correlation, the thicker the line between two nodes.

**Figure 6:**
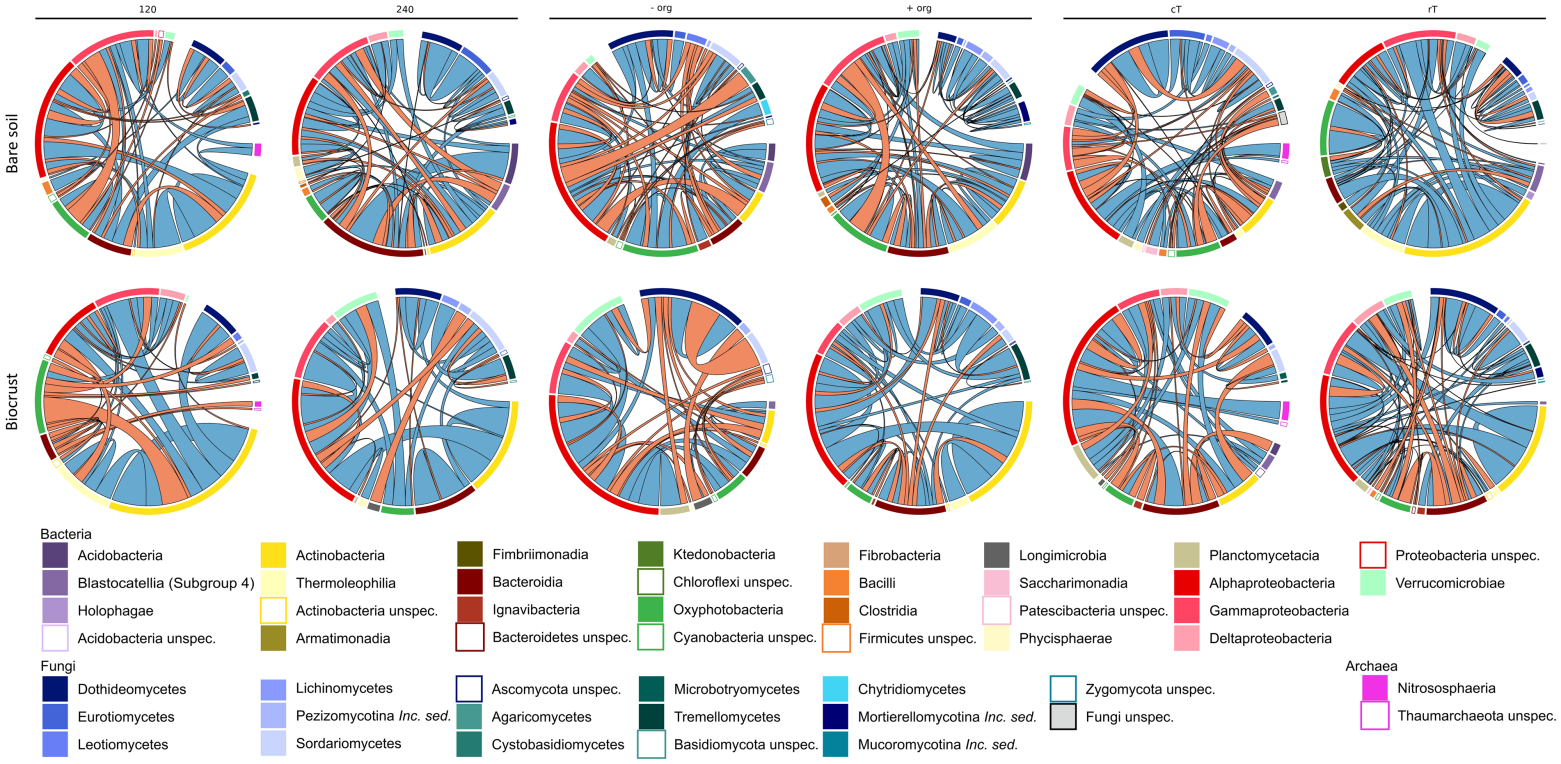
Correlation partners on class level based on the network analysis (*n* = 12) are shown as shares on total edges (in %) for bare soils and biocrusts for each management variation [mineral fertilization amount (120 vs. 240  kg N/ha·a), organic treatment (−org vs. +org), and tillage (conventional (cT) vs. reduced (rT) tillage)]. Red connections display negative and green connections display positive correlation.

Network analysis represents co-occurrence and mutual exclusion patterns of the microbial community based on significant correlations (edges) between taxa (nodes) detected in >80% of the samples. Networks were calculated separately for both compartments (biocrusts and bare soils) and management effects (cT vs. rT, 120 vs. 240 N kg ha^−1^ yr.^−1^, −org vs. +org), resulting in 12 correlation networks based each on 12 samples given in co-occurrence and mutual exclusion (Figures 5, 6). The total number of nodes (in average for bare soils: 209, biocrusts: 152) and edges (bare soils: 367, biocrusts: 238) was higher in bare soils for all treatments (Table 3). Especially rT resulted in the highest numbers of edges and nodes in bare soils reaching 645 and 255, respectively. The same positive effect of rT on the number of edges and nodes was observed for biocrusts (nodes: 223, edges: 417). In biocrusts, the detrimental effect of cT were most pronounced as the number of edges and nodes dropped 1.5 times more compared to bare soils. Regarding the nature of the correlations (co-occurrence vs. mutual exclusion) an interesting pattern emerged based on the comparison of fertilization treatments and tillage: biocrusts had a higher proportion of co-occurrences compared to bare soils in all treatments related to mineral fertilization or organic treatment peaking in the treatments with highest application rates like 240 (77.5%) and + org (76.8%). In contrast, rT and cT networks revealed a higher proportion of co-occurrences in bare soils with the highest value found under rT (77.3%).

**Table 3:** Summary of network parameters including number of nodes and edges per kingdom or between kingdoms and the share of co-occurrences and mutual exclusions.

		Bare soil						Biocrust						120	240	−org	+org	cT	rT	120	240	−org	+org	cT	rT
Total number of ASVs (network input)	Total	4,909	5,438	5,203	5,126	5,189	4,881	4,657	4,395	4,248	4,912	4,172	4,725
	Bacteria	3,941	4,659	4,307	4,320	4,192	4,228	4,122	3,858	3,753	4,357	3,757	4,115
	Archaea	18	11	13	17	15	10	18	13	11	19	14	15
	Fungi	950	768	883	789	982	643	517	524	484	536	401	595
Number of nodes	Total	155	214	163	248	219	255	111	130	128	181	139	223	Shares [%]
	Bacteria	77.42	77.57	66.87	75	56.62	81.57	75.68	73.08	74.22	76.24	79.86	71.3	
	Archaea	1.29	0	0	0	2.74	0.39	3.6	0	0	0	4.32	0	
	Fungi	21.29	22.43	33.13	25	40.64	18.04	20.72	26.92	25.78	23.76	15.83	28.7
Number of edges	Total	193	348	233	448	326	645	173	183	172	320	160	417
Shares [%]	Bacteria - Bacteria	74.6	76.7	48.5	79.9	41.7	88.2	77.5	68.3	63.4	67.2	74.4	69.8
	Archaea - Archaea	0.0	0.0	0.0	0.0	0.0	0.0	0.0	0.0	0.0	0.0	0.0	0.0
	Fungi - Fungi	11.4	17.5	10.3	17.2	25.8	9.6	7.5	16.9	17.4	11.9	6.3	15.6
	Bacteria - Archaea	4.2	0.0	0.0	0.0	6.1	0.2	2.3	0.0	0.0	0.0	7.5	0.0
	Fungi - Archaea	0.0	0.0	0.0	0.0	0.0	0.0	0.0	0.0	0.0	0.0	0.0	0.0
	Bacteria - Fungi	9.8	5.8	41.2	2.9	26.4	2.0	12.7	14.8	19.2	20.9	11.9	14,6
	Co-occurrence	58.2	63.5	51.2	60.4	50.7	77.3	62.9	77.5	50.0	76.8	44.0	65.8
	Mutual exclusion	41.8	36.5	48.8	39.6	49.3	22.7	37.1	22.5	50.0	23.2	56.0	34.2

Bacteria were the dominating kingdom in our network analysis forming up to 82% of nodes. In 11 of 12 networks, the ratio of nodes assigned to Bacteria and Fungi ranged between 2 and 3.6, with cT being the only exception. Here, the Bacteria:Fungi ratio dropped to 1.4 in bare soils, while it strongly increased in biocrusts to a ratio of 5. This could be assigned to a reduction of Tremellomycetes and Dothideomycetes ASVs in the network. With the increasing share of Fungi in the bare soil network, bacterial-fungal correlations increased under cT, mostly between different Basidiomycota and Actinobacteria, Oxyphotobacteria and Phycisphaerae.

In total, 31 different families of Archaea, Bacteria and Fungi in biocrusts and 25 in bare soils were identified as network hubs (highest degree), where 12 families appeared in both compartments as hubs (Figures 5, 6, highlighted in bold in Supplementary Table S12). Out of the 31 hub families, 19 and 13 were specific in biocrusts and bare soils, respectively. Most of these unique hubs only appeared in one or two networks. The only exceptions of the specific families were *Blastocatellaceae* in bare soils, which were identified as hubs in 240, −org, cT, and rT networks and *Rhodobacteraceae* in biocrusts, which formed hubs in networks 120, −org, +org, and cT.

## 4. Discussion

### 4.1. Biocrusts are richer in nutrients and microbial abundance than bare agricultural soils

Biocrusts have previously been described in arid or oligotrophic environments where the accumulation of nutrients is one of their characteristic properties (Brankatschk et al., 2013; Schulz et al., 2013; Maier et al., 2018). To investigate whether this also holds true in fertilized, mesic agricultural ecosystems was one major aim of this study. We observed that under sugar beet cultivation in mesic regions, biocrusts could form large patches (Figure 1; Supplementary Figure S1) with high contents of phototrophic biomass, which was comparable to those of arid biocrusts (Román et al., 2019). Biocrust formation was accompanied by increased DOC, nitrate and DON concentrations as well as higher microbial biomass compared to bare soil implying that these typical biocrust properties are also present in these mesic biocrusts. However, we cannot exclude that this effect changes over the course of a season or at different soil types, which retain nutrients better than these poor soils with high amounts of sand (Kurth et al., 2021). The increase in nutrient concentrations might be attributed to two different mechanisms. First, due to the activity of the phototrophic biomass, CO_2_ is fixed and increased DOC concentrations. This is supported by the positive correlation of DOC (Supplementary Figure S6) and chlorophyll *a* concentration in the biocrusts. Additionally, the significantly higher relative abundance of potential N_2_ fixing bacteria like *Rhizobiaceae* might further facilitate nitrogen input as shown for other biocrusts previously (Lan et al., 2011; Maier et al., 2018; Román et al., 2019). Second, the polymeric matrix of biocrusts traps or retains nutrients more efficiently (Costa et al., 2018; Rossi et al., 2018; Cania et al., 2019a). This is corroborated by the higher relative abundance of Bacteria potentially involved in the production of extracellular polysaccharides in the biocrusts like *Burkholderiaceae*, *Flavobacteriaceae*, *Chitinophagaceae*, *Leptolyngbyaceae*, and *Rhizobiaceae* (Cania et al., 2019a; Vuko et al., 2020).

### 4.2. Biocrusts have less diverse microbial communities but promote co-occurrences across kingdoms

Although microbial biomass and nutrient concentrations were generally higher in biocrusts, alpha-diversity and network density were lower (Figures 3, 5, 6; Supplementary Tables S6, S7). These findings contradict previous biocrust studies, where microbial diversity increased with ongoing biocrust development (Chilton et al., 2018; Maier et al., 2018). However, these studies were conducted in arid or nutrient scarce environments, where biocrusts served as habitable micro-ecosystems and paved the way for the attraction of further microbes. In line with our finding, Glaser et al. (2022b) also found reduced diversity in nutrient-poor, mesic dunes. This data, as well as ours from agricultural biocrusts of mesic regions, support findings from other nutrient-rich hotspots in managed ecosystems like the rhizosphere, drilosphere (Uksa et al., 2015) and biocrusts from mesic forests (Glaser et al., 2022a), where only a subset of the diverse bulk soil community was selected by the specific hotspot. Comparable to those hotspots, correlation networks were dominated by *Sphingomonadaceae* (α-Proteobacteria), *Burkholderiaceae* (γ-Proteobacteria), *Chitinophagaceae* (Bacteroidia), *Nocardioidaceae* (Actinobacteria), and *Pleosporaceae* (Dothideomycetes) (Supplementary Table S12). These families were described as key microbiota during organic matter decomposition (Banerjee et al., 2016), particularly the degradation of chitin and cellulose (Coenye, 2014; Glaeser and Kämpfer, 2014; Maier et al., 2018; Wieczorek et al., 2019). Thus, they may be advantageous for organic C turnover in biocrusts, which were introduced by organic treatment or activities of phototrophic microbes. Furthermore, *Sphingomonadaceae*, *Burkholderiaceae* and *Chitinophagaceae* or *Pleosporaceae* and *Nectriaceae* were further detected as dominating families in exo-and lipopolysaccharides production, especially in developing biocrusts, during soil formation and under conventional tillage (Osińska-Jaroszuk et al., 2015; Xiao and Zheng, 2016; Cania et al., 2019a,b; Vuko et al., 2020). Actinobacteria were important nodes in biocrust networks. However, they were not among the high abundant taxa, which highlights that rarely abundant taxa may contribute essential functions to microbial communities (Shi et al., 2016; Benjamino et al., 2018). Indeed, they were correlated to other proteo-, cyanobacterial or fungal classes but most remarkably was the high proportion of their self-co-occurrences. Though, they share the same niches and promote the growth of other groups of their own phylum (Berry and Widder, 2014; de Vries and Wallenstein, 2017). The actinobacterial network participation was much less under conventional tillage compared to reduced tillage. We observed a drop of 30.7% in bare soils and of 11.6% in biocrusts (see Figures 5, 6). Disturbances were shown to change network composition (Berga et al., 2012) and tillage was shown to affect Actinobacteria due to their hyphal growth form (Cania et al., 2020). Very interesting is that this drop is much lower in biocrusts, which is why we conclude that Actinobacteria are somehow protected against tillage in biocrusts. Important functions covered by Actinobacteria might be their ability to fight fungal infections in crops (Barka et al., 2016) or the degradation of pesticides (Mawang et al., 2021). Further functional analysis would be necessary to answer questions about their beneficial potential in agriculture. Nevertheless, this finding opens the debate for further research about the potential and risks of biocrusts in agricultural ecosystems.

Despite the lower complexity of biocrust correlation networks, the share of co-occurrences was higher in biocrusts compared to bare soil. The polymeric matrix of biocrusts facilitates not only nutrient trapping but also the exchange among microbes by, for example, facilitating movement and horizontal gene transfer interaction among microbes (Costa et al., 2018; Rossi et al., 2018; Cania et al., 2019a). Moreover, the complexity of extracellular polysaccharides and proteins promotes the association of microbes, which can decompose polymers (Rossi et al., 2018). This is further underlined in our study by the finding that the highest frequency of co-occurrences is in the +org treatment, which adds further complex and recalcitrant carbon compounds to the biocrusts (Wang et al., 2017). Indeed, it could be demonstrated earlier that addition of organic treatment increases network complexity in soil (Schmid et al., 2018). The organic amendment specifically increased co-occurrences of bacterial-fungal partners (Figures 5, 6) like Alphaproteobacteria, Dothideomycetes, and Pezizomycotina, which had been identified as keystone taxa in organic matter decomposition previously (Hartmann et al., 2015).

### 4.3. Differential response of Cyanobacteria in bare soil and biocrusts

Cyanobacteria belonged to the dominating Bacteria (Figures 4, 5)—surprisingly, not only in the biocrusts but also in bare soils. This contradicts other agricultural studies where they have not been detected or only displayed a minor proportion of the bulk soil community (Uksa et al., 2015; Cano-Díaz et al., 2019). In particular, *Microcoleus* was one of the abundant observed genera in our study. *Microcoleus* is known as a key taxon for biocrust formation in drylands due to bundle sheath and the production of sticky extracellular polymeric substances (Garcia-Pichel and Wojciechowski, 2009; Pereira et al., 2013) and was shown to attract copiotrophic microbes by releasing photosynthesized carbon into the cyanosphere (Couradeau et al., 2019). This was further accompanied by chlorophyll *a* concentration in the bare soils in the same range as detected for Cyanobacteria-dominated biocrusts in arid areas (Román et al., 2019). Also, we identified Cyanobacteria as network hubs with a proportion of up to 12.5% of all network edges in bare soil networks (Supplementary Table S12), which suggests an early crustal stage, as observed in other early-stage biocrusts (Chilton et al., 2018; Maier et al., 2018). These connections might be beneficial for copiotrophic Bacteria (*Burkholderiaceae*, *Rhodobacteraceae*) and Fungi (*Cucurbitariaceae*), when no additional organic carbon was provided by crop residues (Couradeau et al., 2019; Pombubpa et al., 2020). In contrast, bare soil treatments with organic fertilization promoted negative correlations between Cyanobacteria and Gammaproteobacteria, Dothideo-and Sordariomycetes (Mueller et al., 2015; Wen et al., 2020), which might be attributed to the growth of heterotrophic Bacteria responding to the carbon input (Fierer et al., 2007).

Interestingly, the biocrusts of the cT treatment and the bare soils show similar network pattern with highest cyanobacterial relative abundance and connectivity (Figures 4–6; Supplementary Table S10). As revealed by different studies, soil surface disturbance, like tillage, is a critical factor for biocrust development and sets biocrusts back to an initial development stage, where Cyanobacteria play a crucial role (Lange et al., 1997; Kuske et al., 2012; Bu et al., 2013; Ferrenberg et al., 2015; Steven et al., 2015; Belnap et al., 2016; Maier et al., 2018). Biocrusts that were destroyed by soil turning due to tillage before seeding, quickly re-establish with a fast succession. This regeneration of biocrusts after soil surface disturbance observed in this study happened much faster than observed in (semi-) arid areas (Weber et al., 2016), where recovery rarely takes less than 5 years, and can need several decades depending on the type of disturbance. Nevertheless, to disentangle specific patterns of biocrust establishment on mesic, managed ecosystems, future studies need to analyze disturbance effects in more detail, in more systems and throughout the year.

### 4.4. Conclusion

Our study demonstrates that despite, and sometimes because of, intensive management like tillage and fertilization, biocrusts develop on agricultural fields and build up substantial phototrophic biomass over one growing season. Increased retention of nutrients and water-mediated by biocrusts might improve water and nutrient availability at the beginning of the crop-growing season, possibly with positive feedback on crop growth. This was associated by a reduction of microbial diversity and the promotion of cross-kingdom co-occurrences, indicating that biocrusts become an additional hotspot for activity in soils comparable to rhizosphere or drilosphere. Surprisingly, Cyanobacteria played a negligible role in biocrust networks in autumn before harvest but dominated networks in bare soil communities. Thus, we conclude that they may serve as a seed for biocrust formation, at least in agricultural soils with low quality in terms of organic matter content or waterholding capacity. However, the effect of tillage on the biocrust formation pattern and biocrust properties requires further investigation. Future studies should be accompanied by measurements recording seasonal changes or resilience measurements toward short-term effects like summer drought or heavy rain events.

## Data availability statement

The datasets presented in this study can be found in online repositories. The names of the repository/repositories and accession number(s) can be found in the article, section “Material and Methods”. The raw data for soil properties generated and analyzed for this study will be made available by the corresponding author upon request.

## Author contributions

SS, MS, and UK contributed to the conceptualization and design of this study. MAr manages the fields of LUFA Speyer and assisted in sampling preparation and performance. Sampling was performed by SS. Material preparation, data collection, and analysis were performed by JK. Sequencing was performed by SK. The pipeline for quality filtering of sequencing and taxonomic assignment was provided by CS. The pipeline for network analysis was provided by GV. The first version of the manuscript was written by JK and all authors commented on previous versions of the manuscript. All authors contributed to the article and approved the submitted version.

## Funding

This work has been supported by DFG Nachwuchsakademie (SCHU 2907/4-1) and funded additionally by the BMBF BonaRes project Inplamint (031B1062C). We acknowledge support from the Open Access Publishing Fund of the Technical University Munich.

## Conflict of interest

The authors declare that the research was conducted in the absence of any commercial or financial relationships that could be construed as a potential conflict of interest.

## Publisher’s note

All claims expressed in this article are solely those of the authors and do not necessarily represent those of their affiliated organizations, or those of the publisher, the editors and the reviewers. Any product that may be evaluated in this article, or claim that may be made by its manufacturer, is not guaranteed or endorsed by the publisher.
