# Supplementary material for: Biological soil crusts on agricultural soils of mesic regions promote microbial cross-kingdom co-occurrences and nutrient retention
Source: Front Microbiol. 2023 Jul 14;14:1169958. doi: 10.3389/fmicb.2023.1169958 (PMC10382179; doi:10.3389/fmicb.2023.1169958)
Supplement: Supplementary file 1 [file Data_Sheet_1.pdf]

Supplementary Material

1. Tables

Table S1 Primer, reaction mixture, conditions and calibration standards for real-time qPCR.

Target Region	Reaction conditions<br>thermal profile	No. of Cycles	Calibration standard source	Primer (forward and reverse), Sequence & Reference		BSA (3 %)
16S rRNA gene V5-V6<br>Bacteria	45 s/94 °C, 45 s/58 °C, 45 s/72 °C	40	*Pseudomonas putida*	FP 16S rDNA<br>GGTAGTCYAYGCM-STAAACG<br>(Bach *et al.*, 2002)	0.5	0.5
				RP 16S rDNA<br>GACARCCATGCASCACCTG<br>(Bach *et al.*, 2002)	0.5	
16S rRNA gene V2-V5<br>Archaea	20 s/95 °C, 60 s/55 °C (*), 60 s/72 °C (*) Touchdown -1°C per cycle	5	*Methano-baterium sp.*	SAf (i)<br>CTAYGGGGCGCAGCAGG<br>(Nicol *et al.*, 2003)	0.5	0.5
	20 s/95 °C, 60 s/50 °C, 60 s/72 °C	40		958r<br>YCCGGCGTTGAMTCCAATT<br>(Bano *et al.*, 2003)	0.5	
ITS 1 & 2<br>Fungi	30 s/94 °C, 30 s/50 °C, 30 s/72 °C	40	*Trichoderma viride*	ITS1<br>TCCGTAGGTGAACCTGCGG<br>(White *et al.*, 1990)	1	0
				ITS4<br>TCCTCCGCTTATTGA-TATGC<br>(White *et al.*, 1990)	1	

PCR reactions contained 12.5 µl of Power SybrGreen Master Mix and 2 µl of template DNA plus F- and R-primer (10 µM) and 3 % of bovine serum albumin (BSA) as given in the table (in µl).

Table S2 Primer pairs and sequences, PCR Mastermix and reaction conditions for Amplicon Sequencing.

Gene	Primer (forward and reverse), Sequence & Reference	Mastermix	V [µl]	No. of Cycles	T [°C]	t
16s rRNA Bacteria & Archaea	Arch0519 F CAGCMGCCGCGGTAA (Klindworth *et al.*, 2013)	NebNext High Fidelity Mix	12.5	1	98	5 min
		F- & R primer	0.5		98	20 sec
	Pro 805 R GACTACNVGGGTATCTAATCC (Herlemann *et al.*, 2011)	BSA (3%)	1	25	51	20 sec
		DEPC water	9.5		72	30 sec
				1	72	5 min
ITS Fungi	ITS 3 mix CATCGATGAAGAACGCAG CAACGATGAAGAACGCAG CACCGATGAAGAACGCAG CATCGATGAAGAACGTAG CATCGATGAAGAACGTGG (Tedersoo *et al.*, 2015)	NebNext High Fidelity Mix	10	1	95	15 min
		F- & R primer	0.5		95	30 sec
		BSA (3%)	0	27	55	30 sec
		DEPC water	13		72	60 sec
	ITS 4 mix TCCTCCGCTTATTGATATGC TCCTGCGCTTATTGATATGC TCCTCGCCTTATTGATATGC TCCTCCGCTGAWTAATATGC (Tedersoo *et al.*, 2015)			1	72	10 min

PCR reactions contained 1 µl (3 ng µl^{-1}) of template DNA plus NebtNext Master Mix, F- and R-primer (10 µM), 3 % of bovine serum albumin (BSA) and Diethylpyrocarbonate (DEPC) treated water as given in the table (in µl).

Table S3 Abiotic soil properties of nitrogen (ammonium, nitrate and dissolved organic nitrogen (DON)) and carbon (dissolved organic (DOC) and inorganic (DIC) carbon) pools, chlorophyll *a* (µg per g soil dry weight (dw)), and pH are given as mean values and standard deviation. Treatments are given for tillage (conventional (cT) vs. reduced tillage (rT)), organic treatment (without (-org) vs. with crop residues (+org)), and mineral fertilization amount (120 vs. 240 kg N/ha·a).

Compartment	Treatment	Ammonium		Nitrate		DON		DOC		DIC		Chlorophyll *a*		pH	
		mean	sd	mean	sd	mean	sd	mean	sd	mean	sd	mean	sd	mean	sd
bare soil	pT 120-org	0.52	0.09	7.61	1.90	1.90	0.35	23.25	25.33	5.41	5.06	3.07	1.78	6.14	0.12
bare soil	pT 240 -org	3.02	4.29	17.26	8.84	7.51	8.43	25.51	27.98	8.13	3.86	2.80	0.84	5.90	0.18
bare soil	pT 120+org	0.59	0.17	11.72	0.56	2.26	0.48	8.81	1.91	13.03	4.83	4.63	1.36	6.15	0.07
bare soil	pT 240 +org	0.64	0.04	17.82	3.95	4.02	0.79	14.56	3.48	11.13	4.20	3.85	3.22	6.43	0.06
bare soil	rT 120-org	0.59	0.05	9.61	2.40	1.75	0.32	12.39	1.90	4.24	1.40	3.40	1.69	6.05	0.05
bare soil	rT 240 -org	0.70	0.13	23.22	6.10	4.65	1.67	14.12	1.08	2.64	0.68	3.46	0.70	5.92	0.22
bare soil	rT 120+org	0.58	0.10	16.40	0.82	2.89	1.31	15.36	4.83	3.06	0.93	2.93	0.76	6.20	0.21
bare soil	rT 240 +org	0.54	0.03	21.52	6.15	3.57	0.58	14.54	3.18	5.49	3.84	2.80	0.62	6.12	0.14
biocrust	pT 120-org	1.40	1.26	28.14	17.15	5.73	2.56	18.09	6.73	25.15	17.28	23.28	9.42	6.87	0.11
biocrust	pT 240 -org	2.92	3.68	39.65	9.96	5.09	2.49	19.82	4.82	13.79	5.30	23.16	7.53	6.83	0.10
biocrust	pT 120+org	0.53	0.04	25.55	13.70	4.56	2.24	15.70	5.56	11.32	5.49	17.37	4.60	6.70	0.16
biocrust	pT 240 +org	0.61	0.17	44.95	4.09	7.71	0.92	18.53	3.41	15.78	8.90	25.18	14.58	6.81	0.12
biocrust	rT 120-org	0.70	0.07	31.45	11.98	5.22	2.43	15.87	4.58	8.57	2.50	18.81	8.68	6.62	0.06
biocrust	rT 240 -org	0.69	0.05	37.55	18.57	6.69	5.31	20.40	6.13	10.53	10.02	22.69	15.26	6.68	0.16
biocrust	rT 120+org	0.54	0.13	31.98	10.34	6.27	2.83	16.63	1.92	5.65	1.78	10.95	3.59	6.48	0.03
biocrust	rT 240 +org	0.63	0.31	55.04	16.16	10.87	5.35	29.39	5.26	9.49	5.33	25.32	5.96	6.75	0.17

Table S4 DNA concentration [ng µg-1] as mean ± standard deviation of replicates (n = 3) per treatment, including extraction blanks. Treatments are given for tillage (conventional (cT) vs. reduced tillage (rT)), organic amendments (without (-org) vs. with crop residues (+org)), and mineral fertilization amounts (120 vs. 240 kg N/ha·a).

Compartment		Sample Name	DNA concentration [ng μg^{-1}]
bare soil	cT 120 -org	31,00 ± 11,11	
	cT 240 -org	16,63 ± 8,99	
	cT 120 +org	27,95 ± 3,71	
	cT 240 +org	31,47 ± 18,76	
	rT 120 -org	31,94 ± 4,37	
	rT 240 -org	27,26 ± 1,49	
	rT 120 +org	47,50 ± 4,26	mean of all bare soils
	rT 240 +org	37,73 ± 18,43	31.44
biocrust	cT 120 -org	58,67 ± 4,07	
	cT 240 -org	50,01 ± 16,26	
	cT 120 +org	47,84 ± 11,18	
	cT 240 +org	40,92 ± 7,74	
	rT 120 -org	49,99 ± 14,37	
	rT 240 -org	48,07 ± 6,49	
	rT 120 +org	42,52 ± 10,21	mean of all biocrusts
	rT 240 +org	55,05 ± 6,94	49.13
	extraction blank	0,95 ± 0,77	

Table S5 Copies of target region (per gram soil dry weight (dw)) 16S rRNA gene for Archaea and Bacteria and ITS region for Fungi are given as mean values and standard deviation. Treatments are given for tillage (conventional (cT) vs. reduced tillage (rT)), organic treatment (without (-org) vs. with crop residues (+org)), and mineral fertilization amount (120 vs. 240 kg N/ha·a).

Compartment	Treatment	16S rRNA gene Archaea		16S rRNA gene Bacteria		ITS Fungi	
		mean	sd	mean	sd	mean	sd
bare soil	pT 120-org	30,158,522	28,786,476	15,833,228,596	9,785,044,739	1,209,683,879	501,324,052
bare soil	pT 240 -org	1,115,774	958,086	3,912,995,601	2,268,358,142	13,841,559	7,319,566
bare soil	pT 120+org	4,395,344	2,490,443	6,692,403,534	1,283,100,670	81,588,451	33,006,787
bare soil	pT 240 +org	1,757,812	1,135,305	7,234,086,396	4,298,489,586	678,731,830	585,252,493
bare soil	rT 120-org	877,913	543,421	6,270,253,214	2,329,444,537	9,924,290	6,979,023
bare soil	rT 240 -org	1,055,357	1,273,463	8,518,275,274	4,108,343,309	142,637,751	210,868,316
bare soil	rT 120+org	19,603,592	21,890,057	28,592,677,780	8,498,428,141	1,224,060,890	1,958,259,576
bare soil	rT 240 +org	3,697,594	2,673,294	9,776,191,756	1,245,978,495	1,181,556,224	1,080,255,831
biocrust	pT 120-org	19,479,474	14,160,382	44,179,890,625	8,560,733,258	4,746,620,424	1,326,688,963
biocrust	pT 240 -org	13,931,087	18,283,691	51,267,935,619	34,587,018,705	5,813,208,249	1,442,820,267
biocrust	pT 120+org	22,050,121	20,498,002	26,049,059,757	6,431,184,875	2,035,111,867	372,517,387
biocrust	pT 240 +org	8,196,163	5,786,396	31,974,123,768	7,124,875,252	3,961,607,216	2,250,027,957
biocrust	rT 120-org	18,347,028	4,292,938	32,742,741,321	8,817,402,780	3,675,043,988	372,941,058
biocrust	rT 240 -org	3,866,121	2,928,979	33,264,921,660	11,599,851,124	4,966,968,679	2,752,185,396
biocrust	rT 120+org	22,638,979	13,678,676	24,441,080,206	13,490,605,040	4,708,856,924	4,918,161,917
biocrust	rT 240 +org	4,272,187	3,072,679	37,129,254,298	9,652,908,098	3,960,744,621	2,234,973,563

Table S6 16S rRNA reads for bacterial/archaeal sequencing through pipeline analysis from raw files (fastq) to taxonomy assignment.

			Raw data	Quality filtering						Taxonomy Assignment						
Compart-ment	Sample Name	Repli-cate	Fastq	After Adapter-removal	After Quality Filtering	After Denoising	After Denoising Merged	Without Chimeras	reads lost [%]	16S Without Kingdom Unspec.	Bacteria & Archaea	Chloro-plasts [%]	Mitochon-dria [%]	Bacteria	Bacteria (Phylum unspec.) [%]	Archaea [%]
bare soil	cT 120 -org	a	58985	58959	57279	55266	48976	48871	17.1	48675	48626	5.8	0.2	45460	0.5	0.5
bare soil	cT 120 -org	b	57567	57470	54922	52406	44923	44521	22.7	44435	44423	4.3	0.7	41493	0.2	1.7
bare soil	cT 120 -org	c	50577	50543	49026	47177	42330	42266	16.4	42233	42222	4.7	0.6	39803	0.3	0.4
bare soil	cT 240 -org	a	53140	53087	51312	50047	46563	46287	12.9	45881	45672	25.4	0.5	33788	1.0	0.0
bare soil	cT 240 -org	b	67563	67514	65936	63798	57041	55876	17.3	56697	56661	8.0	0.7	51530	0.1	0.4
bare soil	cT 240 -org	c	113199	113102	109960	107138	98380	97281	14.1	98035	97908	5.6	0.3	91891	0.3	0.2
bare soil	cT 120 +org	a	53549	53484	51790	49709.5	43897	43871	18.1	43729	43611	8.9	0.5	39474	0.5	0.2
bare soil	cT 120 +org	b	65701	65657	63474	60741	52385	52296	20.4	52175	52161	4.0	0.3	49273	0.2	1.4
bare soil	cT 120 +org	c	62575	62520	60556	57849	48920	48663	22.2	48632	48559	7.7	0.3	44358	0.7	0.7
bare soil	cT 240 +org	a	70473	70362	67982	65852	58905	58704	16.7	58611	58559	6.3	0.2	54694	0.4	0.1
bare soil	cT 240 +org	b	87981	87936	85382	82744	73603	72765	17.3	72817	72770	5.4	0.3	68519	0.2	0.2
bare soil	cT 240 +org	c	64374	64213	62301	59489	50226	50111	22.2	50023	50003	3.4	0.3	47658	0.1	1.0
bare soil	rT 120 -org	a	98663	98519	95095	92360	82864	82291	16.6	81903	81732	12.6	0.6	70633	0.7	0.5
bare soil	rT 120 -org	b	78409	78294	75914	73582	66158	65978	15.9	65715	65662	7.4	0.4	60351	0.3	0.3
bare soil	rT 120 -org	c	41321	41265	40064	38078	32143	32067	22.4	31963	31955	6.5	0.3	29654	0.4	0.5
bare soil	rT 240 -org	a	267150	266923	259562	255710	241211	234062	12.4	238655	238353	9.4	0.6	214327	0.6	0.1
bare soil	rT 240 -org	b	50560	50520	47479	45229	35467	35467	29.9	35286	35269	11.0	0.8	31084	0.6	0.0
bare soil	rT 240 -org	c	72707	72536	70204	67999	60864	60661	16.6	60538	60499	9.4	0.5	54449	0.5	0.1
bare soil	rT 120 +org	a	82287	82284	60984	54654	27901	27668	66.4	27555	27543	8.4	0.5	25016	0.2	0.3
bare soil	rT 120 +org	b	77634	77520	74846	71908.5	62047	61862	20.3	61702	61638	10.0	0.5	54727	0.7	0.9

bare soil	rT 120 +org	c	73037	72978	70897	67920	57595	57385	21.4	57200	57027	8.9	0.4	51308	0.5	0.9
bare soil	rT 240 +org	a	62427	62342	60526	58011	50536	50272	19.5	50218	50141	3.6	0.4	48047	0.2	0.2
bare soil	rT 240 +org	b	78846	78800	75913	73191	62843	62615	20.6	62300	62158	13.2	1.0	53189	0.6	0.3
bare soil	rT 240 +org	c	66239	66148	64391	61870	53826	53423	19.3	53192	53018	10.2	0.5	47149	0.6	0.4
biocrust	cT 120 -org	a	71163	71122	68267	66175	58429	58150	18.3	57995	57852	7.2	0.5	53279	0.4	0.1
biocrust	cT 120 -org	b	75375	75315	73053	70388	62857	62451	17.1	62327	62284	5.5	0.3	58327	0.3	0.6
biocrust	cT 120 -org	c	54951	54908	53189	51435.5	47011	46938	14.6	46821	46796	6.4	0.2	43598	0.5	0.2
biocrust	cT 240 -org	a	57736	57690	55433	53991	48829	48578	15.9	48278	48189	10.9	0.0	42881	0.6	0.0
biocrust	cT 240 -org	b	49763	49710	48149	45906	39937	39848	19.9	39775	39737	8.2	0.2	36202	0.4	0.6
biocrust	cT 240 -org	c	46869	46796	45317	43597	39166	39112	16.6	38934	38873	13.0	0.5	33626	0.4	0.1
biocrust	cT 120 +org	a	47105	47068	45809	43895	38748	38337	18.6	38227	38119	13.4	1.0	32571	0.4	0.2
biocrust	cT 120 +org	b	62340	62278	60542	58267	51669	51606	17.2	51520	51502	10.7	0.5	45310	0.2	1.0
biocrust	cT 120 +org	c	52723	52620	50879	48744	42488	42264	19.8	42159	42030	10.4	0.4	37364	1.8	0.3
biocrust	cT 240 +org	a	203856	203799	198520	194518	181574	178235	12.6	180265	179930	8.9	0.5	162670	0.5	0.2
biocrust	cT 240 +org	b	49626	49588	47940	46006	40390	40234	18.9	40116	40066	6.9	0.2	37184	0.3	0.1
biocrust	cT 240 +org	c	79731	79687	77058	74709	67621	67227	15.7	67142	67074	5.7	0.1	63016	0.3	0.3
biocrust	rT 120 -org	a	76639	76448	73304	70894	64226	63963	16.5	63653	63478	15.3	1.2	52700	1.4	0.6
biocrust	rT 120 -org	b	60751	60679	58669	56624	51023	50937	16.2	50650	50562	17.8	1.4	40834	1.1	0.0
biocrust	rT 120 -org	c	71298	71210	69049	66835	61002	60909	14.6	60561	60512	21.8	0.4	46812	1.8	0.5
biocrust	rT 240 -org	a	63397	63319	61068	59617	55614	55614	12.3	55232	55099	28.3	1.2	38799	1.7	0.0
biocrust	rT 240 -org	b	50143	50081	48803	47177	42769	42712	14.8	42287	42217	19.4	0.9	33650	0.5	0.0
biocrust	rT 240 -org	c	76220	76178	74209	71638	63157	62894	17.5	62450	62425	14.7	0.2	52979	0.3	0.3
biocrust	rT 120 +org	a	245145	244978	238278	232951	213831	208235	15.1	211255	210921	10.7	0.3	186111	0.3	0.8
biocrust	rT 120 +org	b	57148	57063	55158	52389	43661	43520	23.8	43303	43244	10.8	0.3	38099	0.3	0.9
biocrust	rT 120 +org	c	52033	51926	50178	47804	41101	40954	21.3	40815	40693	14.2	0.8	34429	0.5	0.5

biocrust	rT 240 +org	a	41050	41016	39631	38096	34055	33953	17.3	33411	33288	37.2	1.5	20405	1.7	0.0
biocrust	rT 240 +org	b	51582	51537	49737	47809	42866	42816	17.0	42384	42231	37.8	0.8	25904	1.7	0.1
biocrust	rT 240 +org	c	50008	49958	48107	46465	41800	41700	16.6	41509	41363	15.3	0.2	34949	0.8	0.1
	extraction blank		167	122	46	1.5	0	0	100.0	0	0	0.0	0.0	0	0.0	0.0
	extraction blank		173	97	44	6	5	5	97.1	5	5	0.0	0.0	5	0.0	0.0
	extraction blank		319	197	174	75.5	64	64	79.9	64	64	9.4	0.0	58	0.0	0.0
	extraction blank		104	81	68	35	35	35	66.3	35	35	0.0	0.0	35	0.0	0.0
	PCR NTC		106	18	9	1	0	0	100.0	0	0	0.0	0.0	0	0.0	0.0
	PCR NTC		107	68	8	1	0	0	100.0	0	0	0.0	0.0	0	0.0	0.0
	PCR NTC		184	169	15	1.5	0	0	100.0	0	0	0.0	0.0	0	0.0	0.0
	PCR NTC		111	49	12	1	0	0	100.0	0	0	0.0	0.0	0	0.0	0.0

Table S7 ITS reads for fungal sequencing through pipeline analysis from raw files (fastq) to taxonomy assignment.

			Raw data	Quality filtering						Taxonomy Assignment		
Compartment	Sample Name	Repli-cate	Fastq	After Adapter-removal	After Quality Filtering	After Denoising	After Denoising Merged	Without Chimeras	reads lost [%]	ITS Without Kingdom Unspec.	Fungi	Fungi (Phylum unspec.) [%]
bare soil	cT 120 -org	a	110253	108159	95108	93742	88926	77820	28.1	77754	75005	39.2
bare soil	cT 120 -org	b	237638	232879	203872	201766	194726	164838	29.2	159177	150984	40.7
bare soil	cT 120 -org	c	107262	106297	92048	90505	81502	71115	33.1	70154	66132	46.1
bare soil	cT 240 -org	a	256417	255102	232995	231861	227239	191363	25.0	191358	186141	31.3
bare soil	cT 240 -org	b	280742	275427	239576	237741	228054	189210	31.3	183131	175637	57.4
bare soil	cT 240 -org	c	167366	164228	143771	142153	136093	111948	31.8	110747	108171	57.2
bare soil	cT 120 +org	a	146038	144576	124040	122535	113998	96907	33.0	93668	91745	56.9
bare soil	cT 120 +org	b	203931	201300	175342	173449	164695	139215	30.8	127546	121696	43.1
bare soil	cT 120 +org	c	190005	187025	165575	163551	152907	130693	30.1	128283	124342	29.6
bare soil	cT 240 +org	a	72451	72065	63941	62976	60751	53511	25.7	54049	52831	29.3
bare soil	cT 240 +org	b	113833	113063	101810	100412	96401	82782	26.8	82125	75092	34.2
bare soil	cT 240 +org	c	170995	168851	147455	145914	138057	111873	33.7	114080	96514	22.7
bare soil	rT 120 -org	a	92166	90433	80481	79264	75006	64817	28.3	64794	62889	55.8
bare soil	rT 120 -org	b	62790	62293	55755	54533	51945	46979	24.6	45588	44777	26.3
bare soil	rT 120 -org	c	82956	82376	72732	71433	67588	59360	27.9	59279	57675	46.5
bare soil	rT 240 -org	a	101438	100716	91361	90354	86793	73506	27.0	73502	65553	35.9
bare soil	rT 240 -org	b	158627	157801	146359	144584	137487	120147	23.9	121010	117387	44.4
bare soil	rT 240 -org	c	80779	80385	71559	70367	67857	58978	26.6	58978	57400	45.5
bare soil	rT 120 +org	a	76991	76476	67502	66144	61959	56049	26.7	55856	54744	22.4
bare soil	rT 120 +org	b	62542	61619	55033	53676	50083	44511	27.8	44223	42183	42.9

bare soil	rT 120 +org	c	55525	55064	49323	48277	45342	39765	27.8	39751	38610	54.3
bare soil	rT 240 +org	a	43844	43527	39502	38482	35622	32364	25.6	32362	30686	31.1
bare soil	rT 240 +org	b	51264	50929	45191	44197	41823	37359	26.6	37359	36839	41.8
bare soil	rT 240 +org	c	51907	51350	46252	45160	42760	37844	26.3	37989	37642	33.7
biocrust	cT 120 -org	a	49739	49464	44189	43356	41336	36433	26.3	36376	35973	38.4
biocrust	cT 120 -org	b	33610	33417	29481	28685	27223	24303	27.3	23979	23892	57.9
biocrust	cT 120 -org	c	45401	45246	38134	37133	34815	31580	30.2	31840	31481	40.6
biocrust	cT 240 -org	a	47948	47279	42551	41973	40898	33386	29.4	33386	30256	38.0
biocrust	cT 240 -org	b	61516	61100	54907	54184	52434	45349	25.8	45235	43314	36.1
biocrust	cT 240 -org	c	47958	47585	42347	41419	38843	34107	28.3	34019	33433	59.0
biocrust	cT 120 +org	a	49041	48858	41805	40994	35078	30005	38.6	29558	29311	82.0
biocrust	cT 120 +org	b	99630	99229	85276	83886	75229	66558	32.9	62665	61405	59.6
biocrust	cT 120 +org	c	46588	46248	38186	37052	33730	30940	33.1	30517	30081	49.8
biocrust	cT 240 +org	a	106824	105597	94348	93058	83625	70841	32.9	70440	68895	63.6
biocrust	cT 240 +org	b	84844	84524	75943	74809	67494	58494	30.8	57341	56136	75.8
biocrust	cT 240 +org	c	115663	115126	104071	103051	97807	81247	29.4	81206	80219	61.0
biocrust	rT 120 -org	a	99687	98666	87695	86414	81565	68238	30.8	68191	66480	67.0
biocrust	rT 120 -org	b	119963	119059	107335	105693	98848	84457	29.1	83919	81789	50.7
biocrust	rT 120 -org	c	125528	124016	108144	106490	101462	84987	31.5	84231	82217	44.5
biocrust	rT 240 -org	a	94283	93544	83589	82586	77846	65398	30.1	65306	61718	68.9
biocrust	rT 240 -org	b	85443	84845	75636	74555	69023	59764	29.6	59678	57535	57.0
biocrust	rT 240 -org	c	107944	107527	95580	94201	89999	76848	28.5	78275	77490	29.1
biocrust	rT 120 +org	a	100747	100358	90455	89258	83397	73332	26.9	72204	70179	47.5
biocrust	rT 120 +org	b	99699	99387	89306	88014	80753	70532	29.0	70506	69221	61.3
biocrust	rT 120 +org	c	101200	100037	89406	88049	83932	69380	30.6	69370	68135	48.4

biocrust	rT 240 +org	a	92574	88405	79214	78074	73846	58867	33.4	58854	57389	77.2
biocrust	rT 240 +org	b	103593	102297	89346	87760	81417	64663	36.8	64613	63258	72.5
biocrust	rT 240 +org	c	98446	95343	84287	83145	78571	63383	33.5	63537	62312	53.8
	extraction blank		9425	9390	1612	1582	1579	1253	86.7	1253	1253	0.0
	extraction blank		5075	4990	53	16	11	11	99.8	11	11	36.4
	extraction blank		135	133	15	1	0	0	100.0	0	0	0.0
	extraction blank		14470	13918	45	17	6	6	100.0	6	6	100.0
	PCR NTC		7558	7260	27	3	3	3	100.0	3	3	100.0
	PCR NTC		17833	1424	5	2	2	2	99.9	2	2	0.0
	PCR NTC		3795	3143	19	5	3	3	99.9	3	3	100.0
	PCR NTC		8201	5487	21	4	0	0	100.0	0	0	0.0

Table S8 Alpha diversity for prokaryotic 16S rRNA and ITS Fungi for Shannon diversity (S), richness (R) and Pielou's eveness (P) as mean ± standard deviation. Treatments are given for tillage (conventional (cT) vs. reduced tillage (rT)), organic amendments (without (-org) vs. with crop residues (+org)), and mineral fertilization amounts (120 vs. 240 kg N/ha·a)

Gene	Compartment	Tillage	Mineral Fertilization Amounts	Organic Amendments	S	R	P
Prokaryotic 16S rRNA	bare soil	cT	120	-org	5.90 ± 0.46	890 ± 118.1	0.869 ± 0.053
				+org	6.02 ± 0.43	1003 ± 170.7	0.872 ± 0.041
			240	-org	5.92 ± 0.13	1038 ± 383.0	0.859 ± 0.03
				+org	6.32 ± 0.15	1138 ± 72.5	0.898 ± 0.018
		rT	120	-org	6.02 ± 0.38	1037.7 ± 306.1	0.871 ± 0.018
				+org	6.09 ± 0.87	946.3 ± 628.2	0.926 ± 0.015
			240	-org	6.32 ± 0.48	1314.7 ± 795.2	0.898 ± 0.017
				+org	6.48 ± 0.06	1153.3 ± 34.5	0.919 ± 0.005
	biocrust	cT	120	-org	5.93 ± 0.31	928.7 ± 168	0.869 ± 0.023
				+org	5.81 ± 0.35	820.3 ± 150.9	0.866 ± 0.032
			240	-org	5.93 ± 0.02	797.3 ± 42.1	0.888 ± 0.008
				+org	6.13 ± 0.42	1245.7 ± 593.1	0.868 ± 0.007
		rT	120	-org	6.05 ± 0.03	965.7 ± 46.7	0.88 ± 0.009
				+org	6.48 ± 0.36	1447.7 ± 845.4	0.903 ± 0.017
			240	-org	6.11 ± 0.36	939 ± 287.9	0.896 ± 0.015
				+org	5.91 ± 0.01	677 ± 69.8	0.908 ± 0.016
ITS Fungi	bare soil	cT	120	-org	4.66 ± 0.25	512.3 ± 107.9	0.748 ± 0.028
				+org	4.80 ± 0.27	537 ± 96.4	0.764 ± 0.029

		240	-org	3.93 ± 0.92	438.7 ± 137.3	0.645 ± 0.119
			+org	3.98 ± 0.30	327.7 ± 86.4	0.691 ± 0.049
	rT	120	-org	4.28 ± 0.24	339.7 ± 23.1	0.735 ± 0.038
			+org	4.50 ± 0.08	332.7 ± 41.7	0.776 ± 0.008
		240	-org	4.22 ± 0.33	332.7 ± 74	0.727 ± 0.03
			+org	4.44 ± 0.08	303.7 ± 7.8	0.776 ± 0.011
biocrust	cT	120	-org	3.71 ± 0.23	167.7 ± 18.9	0.725 ± 0.042
			+org	3.98 ± 0.26	238.7 ± 93.9	0.733 ± 0.003
		240	-org	3.52 ± 0.53	162.7 ± 48.4	0.694 ± 0.065
			+org	4.02 ± 0.29	302.7 ± 55.2	0.705 ± 0.030
	rT	120	-org	4.33 ± 0.19	387.7 ± 47	0.728 ± 0.017
			+org	4.17 ± 0.25	345.3 ± 14.8	0.713 ± 0.038
		240	-org	4.11 ± 0.28	303.7 ± 94.5	0.722 ± 0.019
			+org	3.94 ± 0.30	286 ± 24.9	0.696 ± 0.044

Table S9 p and F values of linear models for alpha diversity of 16S rRNA Bacteria/Archaea and ITS Fungi as Shannon diversity (S), richness (R) and Pielou's eveness (P). Significant values (p ≤ 0.05) are marked in bold.

Gene	Variable	Biocrust effect		Compartment	Tillage		Mineral Fertilizer Amount		Organic Amendments		Tillage * Organic Amendments		Tillage * Mineral Fertilizer Amount		Mineral Fertilizer Amount * Organic Amendments		Tillage * Mineral Fertilizer Amount * Organic Amendments	
		F	p		F	p	F	p	F	p	F	p	F	p	F	p	F	p
Prokaryotic 16S rRNA	S	0.60	0.441	bare soil	0.90	0.356	0.92	0.351	1.94	0.182	0.19	0.666	0.26	0.617	0.30	0.590	0.04	0.846
				biocrust	2.60	0.126	0.35	0.565	0.12	0.736	0.13	0.727	3.18	0.094	0.39	0.543	3.87	0.067
	R	0.45	0.506	bare soil	0.00	0.974	0.01	0.929	1.19	0.291	0.56	0.466	0.21	0.656	0.22	0.649	0.16	0.694
				biocrust	0.06	0.805	0.29	0.599	1.13	0.303	0.29	0.600	3.94	0.065	0.05	0.830	5.54	**0.032**
	P	0.21	0.647	bare soil	6.02	**0.026**	6.10	**0.025**	0.60	0.451	0.48	0.497	0.00	0.949	0.00	0.968	2.24	0.154
				biocrust	10.63	**0.005**	0.17	0.688	2.02	0.174	3.94	0.065	0.01	0.941	1.03	0.326	0.05	0.820
ITS Fungi	S	8.82	**0.005**	bare soil	0.10	0.755	0.96	0.342	5.86	**0.028**	0.06	0.804	4.20	0.057	0.00	0.983	0.00	0.993
				biocrust	6.66	**0.020**	0.90	0.357	1.44	0.248	4.72	**0.045**	0.23	0.635	0.24	0.632	0.29	0.599
	R	16.33	**0.000**	bare soil	11.94	**0.003**	0.94	0.346	5.65	**0.030**	0.14	0.709	2.78	0.115	1.27	0.276	0.68	0.422
				biocrust	24.64	**0.000**	4.77	**0.044**	0.44	0.516	8.77	**0.009**	3.32	0.087	1.24	0.281	0.47	0.501
	P	1.08	0.304	bare soil	3.57	0.077	2.86	0.110	4.34	0.054	0.03	0.876	3.72	0.072	0.27	0.613	0.14	0.716
				biocrust	0.01	0.940	0.11	0.749	1.85	0.193	1.04	0.323	0.38	0.545	0.02	0.888	0.06	0.803

Table S10 p and F values of highly abundant bacterial families (at least 2% per replicate) reacting on interacting management effects. Families shown in bold are affected in bare soil and biocrust. Significant effects (p < 005) are shown in bold.

Compart-ment	Taxa	Tillage		Mineral Fertilizer Amount		Organic Amendments		Tillage * Organic Amend-ments		Tillage * Mineral Fertilizer Amount		Mineral Fertilizer Amount * Organic Amend-ments		Tillage * Mineral Fertilizer Amount * Organic Amendments	
		F	p	F	p	F	p	F	p	F	p	F	p	F	p
bare soil	**k__Bacteria, p__Bacteroidetes, c__Bacteroidia, o__Sphingobacteriales, f__env.OPS_17**	0.27	0.608	4.41	0.052	1.06	0.318	0.37	0.552	0.66	0.430	3.29	0.088	5.96	**0.027**
	k__Bacteria, p__Cyanobacteria, c__Oxyphotobacteria, o__Leptolyngbyales, f__Leptolyngbyaceae	0.57	0.463	1.58	0.226	1.65	0.217	6.10	**0.025**	0.08	0.785	0.08	0.780	0.35	0.562
	k__Bacteria, p__Proteobacteria, c__Gammaproteobacteria, o__Alteromonadales, f__Alteromonadaceae	1.60	0.223	1.16	0.297	4.85	**0.043**	4.74	**0.045**	2.04	0.172	1.54	0.232	4.05	0.061
	k__Bacteria, p__Proteobacteria, c__Gammaproteobacteria, o__Xanthomona-dales, f__Xanthomonadaceae	0.34	0.569	37.13	**0.000**	26.29	**0.000**	0.40	0.535	11.87	**0.003**	0.84	0.373	6.20	**0.024**
	**k__Bacteria, p__Verrucomicrobia, c__Verrucomicrobiae, o__Chthoniobacterales, f__Chthoniobacteraceae**	5.40	**0.034**	3.86	0.067	7.23	**0.016**	0.70	0.416	0.37	0.552	0.50	0.492	4.87	**0.042**
biocrust	k__Bacteria, p__Actinobacteria, c__Actinobacteria, o__Propionibacteriales, f__Nocardioidaceae	5.62	**0.031**	4.47	0.051	5.91	**0.027**	0.86	0.367	7.08	**0.017**	0.26	0.615	7.12	**0.017**
	k__Bacteria, p__Actinobacteria, c__Actinobacteria, o__Pseudonocardiales, f__Pseudonocardiaceae	2.96	0.105	5.10	0.038	5.89	**0.027**	0.32	0.580	13.98	**0.002**	0.34	0.565	12.02	**0.003**
	k__Bacteria, p__Bacteroidetes, c__Bacteroidia, o__Chitinophagales, f__Chitinophagaceae	23.03	**0.000**	0.27	0.609	6.75	**0.019**	2.54	0.130	0.90	0.356	5.42	**0.033**	6.59	**0.021**
	**k__Bacteria, p__Bacteroidetes, c__Bacteroidia, o__Sphingobacteriales, f__env.OPS_17**	7.81	**0.013**	0.76	0.395	2.40	0.141	**0.03**	0.872	4.98	**0.040**	0.53	0.476	6.45	**0.022**
	k__Bacteria, p__Bacteroidetes, c__Bacteroidia, o__Sphingobacteriales, f__Sphingobacteriaceae	13.54	**0.002**	**0.01**	0.908	8.62	**0.010**	5.17	**0.037**	3.24	0.091	1.83	0.195	3.41	0.083
	k__Bacteria, p__Cyanobacteria, c__Oxyphotobacteria, o__Oxyphotobacteria_Incertae_Sedis, f__Unknown_Family	7.95	**0.012**	3.97	0.064	1.65	0.218	5.65	**0.030**	0.75	0.400	2.55	0.130	0.43	0.521
	k__Bacteria, p__Gemmatimonadetes, c__Longimicrobia, o__Longimicrobiales, f__Longimicrobiaceae	3.82	0.068	0.06	0.811	**0.00**	0.970	4.44	0.051	5.96	**0.027**	0.64	0.435	2.74	0.118
	k__Bacteria, p__Proteobacteria, c__Alphaproteobacteria, o__Rhizobiales, f__Beijerinckiaceae	20.84	**0.000**	0.22	0.644	8.63	**0.010**	0.05	0.821	4.56	**0.048**	0.01	0.919	5.34	**0.035**
	k__Bacteria, p__Proteobacteria, c__Deltaproteobacteria, o__Myxococcales, f__Archangiaceae	12.72	**0.003**	0.50	0.490	7.71	**0.013**	2.43	0.139	8.44	**0.010**	4.47	0.051	17.38	**0.001**
	**k__Bacteria, p__Verrucomicrobia, c__Verrucomicrobiae, o__Chthoniobacterales, f__Chthoniobacteraceae**	4.70	**0.046**	4.71	**0.045**	30.36	**0.000**	4.46	0.051	2.20	0.157	5.68	**0.030**	2.21	0.156
	k__Bacteria, p__Verrucomicrobia, c__Verrucomicrobiae, o__Verrucomicrobiales, f__Rubritaleaceae	0.54	0.475	5.14	0.038	30.33	**0.000**	2.86	0.110	12.75	**0.003**	0.06	0.805	0.17	0.685

Table S11 p and F values of highly abundant fungal families (at least 2% per replicate) reacting on interacting management effects. Families shown in bold are affected in bare soil and biocrust. Significant effects (p < 005) are shown in bold.

Compart-ment	Taxa	Tillage		Mineral Fertilizer Amount		Organic Amendments		Tillage * Organic Amendments		Tillage * Mineral Fertilizer Amount		Mineral Fertilizer Amount * Organic Amendments		Tillage * Mineral Fertilizer Amount * Organic Amendments	
		F	p	F	p	F	p	F	p	F	p	F	p	F	p
bare soil	k__Fungi, p__Ascomycota, c__Dothideomycetes, o__Pleosporales, f__Pleosporales_fam_Incertae_sedis	**0.00**	0.987	2.65	0.123	1.84	0.194	7.92	**0.012**	1.52	0.236	0.02	0.900	2.78	0.115
	**k__Fungi, p__Ascomycota, c__Leotiomycetes, o__Helotiales, f__Helotiales_fam_Incertae_sedis**	11.76	**0.003**	20.79	**0.000**	2.36	0.144	13.70	**0.002**	1.91	0.186	0.14	0.712	0.85	0.370
	k__Fungi, p__Ascomycota, c__Lichinomycetes, o__Lichinales, f__Lichinaceae	11.20	**0.004**	7.82	**0.013**	1.15	0.300	8.31	**0.011**	0.72	0.408	0.09	0.767	0.08	0.781
	k__Fungi, p__Ascomycota, c__Sordariomycetes, o__Sordariales, f__Lasiosphaeriaceae	5.10	**0.038**	12.27	**0.003**	9.04	**0.008**	0.13	0.718	4.88	**0.042**	1.81	0.197	0.37	0.551
	k__Fungi, p__Basidiomycota, c__Cystobasidiomycetes, o__Cystobasidiomy-cetes_ord_Incertae_sedis, f__Cystobasidiomycetes_fam_Incertae_sedis	5.30	**0.035**	3.35	0.086	3.80	0.069	2.23	0.155	1.65	0.217	6.01	**0.026**	5.26	**0.036**
	k__Fungi, p__Basidiomycota, c__Tremellomycetes, o__Cystofilobasidiales, f__Cystofilobasidiaceae	14.14	**0.002**	13.45	**0.002**	0.26	0.615	7.90	**0.013**	0.86	0.367	0.92	0.352	1.38	0.257
	k__Fungi, p__Basidiomycota, c__Tremellomycetes, o__Filobasidiales, f__Piskurozymaceae	5.31	**0.035**	6.27	**0.023**	5.74	**0.029**	6.89	**0.018**	0.81	0.381	0.41	0.530	0.06	0.804
	**k__Fungi, p__Basidiomycota, c__Tremellomycetes, o__Tremellales, f__Tremellales_fam_Incertae_sedis**	3.25	0.090	0.71	0.411	5.41	**0.034**	11.62	**0.004**	0.07	0.794	0.46	0.506	0.80	0.384
	k__Fungi, p__Zygomycota, c__Mucoromycotina_cls_Incertae_sedis, o__Mucorales, f__Mucoraceae	0.12	0.731	3.56	0.077	0.12	0.732	5.51	**0.032**	2.33	0.147	1.07	0.316	3.55	0.078
biocrust	k__Fungi, p__Ascomycota, c__Dothideomycetes, o__Pleosporales, f__Cucurbitariaceae	7.06	**0.017**	0.38	0.545	1.08	0.314	17.26	**0.001**	3.25	0.090	0.00	0.979	0.02	0.878
	k__Fungi, p__Ascomycota, c__Dothideomycetes, o__Pleosporales, f__Pleosporaceae	5.09	**0.038**	1.59	0.226	4.37	0.053	0.86	0.367	6.12	**0.025**	0.00	0.961	0.00	0.953
	k__Fungi, p__Ascomycota, c__Eurotiomycetes, o__Chaetothyriales, f__Herpotrichiellaceae	9.67	**0.007**	6.97	**0.018**	0.38	0.544	5.36	**0.034**	3.12	0.097	0.09	0.765	4.42	0.052
	**k__Fungi, p__Ascomycota, c__Leotiomycetes, o__Helotiales, f__Helotiales_fam_Incertae_sedis**	36.69	**0.000**	32.78	**0.000**	8.75	**0.009**	9.71	**0.007**	0.83	0.377	0.18	0.680	0.45	0.514
	k__Fungi, p__Ascomycota, c__Pezizomycotina_cls_Incertae_sedis, o__Pezizomycotina_ord_Incertae_sedis, f__Pezizomycotina_fam_Incertae_sedis	20.38	**0.000**	1.22	0.285	0.84	0.372	2.06	0.170	5.83	**0.028**	1.05	0.320	0.01	0.913
	k__Fungi, p__Ascomycota, c__Sordariomycetes, o__Hypocreomyceti-dae_ord_Incertae_sedis, f__Plectosphaerellaceae	2.29	0.150	3.60	0.076	0.37	0.549	1.19	0.292	5.37	**0.034**	0.50	0.492	0.44	0.518
	k__Fungi, p__Ascomycota, c__Sordariomycetes, o__Sordariales, f__Chaetomiaceae	8.24	**0.011**	**0.00**	0.961	5.64	**0.030**	6.31	**0.023**	7.66	**0.014**	0.71	0.411	0.09	0.773
	k__Fungi, p__Ascomycota, c__Sordariomycetes, o__Sordariomyceti-dae_ord_Incertae_sedis, f__Glomerellaceae	**0.01**	0.939	5.00	**0.040**	0.10	0.757	0.22	0.647	5.65	**0.030**	1.36	0.261	0.50	0.490
	**k__Fungi, p__Basidiomycota, c__Tremellomycetes, o__Tremellales, f__Tremellales_fam_Incertae_sedis**	4.85	**0.043**	24.87	**0.000**	5.02	**0.040**	2.70	0.120	0.52	0.481	10.93	**0.004**	0.43	0.521

Table S12 Top 10 network hubs as most connected families for each network separately. Network participation is given in percentage of degree of total edges and share on positive edges. Hubs not specified at the family level were ignored. Common ones in both sample types (bare soil and biocrust) are shown in bold. Missing numbers indicate that these families either do not appear in a certain network or are not below the Top 10 hubs within this network.

Compart-ment	Taxa			120		240		-org		+org		cT		rT	
	Phylum	Class	Family	total degree [%]	positive edges [%]	total degree [%]	positive edges [%]	total degree [%]	positive edges [%]	total degree [%]	positive edges [%]	total degree [%]	positive edges [%]	total degree [%]	positive edges [%]
Bare soil	Acido-bacteria	Acidobacteria	Acidobacteriaceae (Subgroup 1)			5.2	5.2			4.9	4.9				
		Blastocatellia (Subgroup 4)	Blastocatellaceae			8.9	1.2	9.9	8.2			6.1	3.7	8.8	6.1
	Actino-bacteria	Actinobacteria	**Intrasporangiaceae**	6.7	6.7	4.3	3.5							7.4	6.8
			**Micrococcaceae**	10.9	10.9							5.5	0.0		
			**Nocardioidaceae**	9.3	7.8	10.9	7.8	7.3	3.4	7.1	5.8			17.8	16.4
		Thermo-leophilia	**Gaiellaceae**											5.9	5.9
	Asco-mycota	Dothideomycetes	Cucurbitariaceae					8.2	6.4						
			**Pleosporaceae**	5.7	4.2							7.4	4.9	5.6	2.0
		Eurotiomycetes	Herpotrichiellaceae			9.2	9.2								
		Sordariomycetes	**Nectriaceae**	5.7	4.2	5.5	4.6								
			Plectos-phaerellaceae									6.1	3.7		
	Bacte-roidetes	Bacteroidia	**Chitinophagaceae**	8.3	3.1	26.2	16.7	11.6	5.6	11.2	6.9	5.5	3.7	8.1	8.1
			env.OPS_17	6.2	6.2					6.0	4.0				
	Cyano-bacteria	Oxyphoto-bacteria	Leptolyngbyaceae					6.4	4.7					5.6	4.0
			**“Unknown Family”**	8.8	3.1	4.3	2.9	12.5	11.6	9.4	4.5	7.1	3.1	10.2	9.9
			Devosiaceae					6.4	1.7						

	Proteobacteria	Alphaproteobacteria	Hyphomicrobiaceae							5.8	5.1				
			Methyloligellaceae									8.6	8.6		
			**Rhodobacteraceae**									5.5	0.0		
			**Sphingomonadaceae**	19.2	12.4	16.4	14.9	24.9	13.3	21.0	17.2			14.6	13.3
		Gammaproteobacteria	**Burkholderiaceae**	21.2	10.9	10.1	5.2	8.2	3.4	12.7	3.6	8.9	0.0	9.2	7.1
			Nitrosomonadaceae							6.9	4.9				
			**Xanthomonadaceae**					7.7	3.0						
	Verrucomicrobia	Verrucomicrobiae	Verrucomicrobiaceae									6.1	3.7		
	Zygomycota	Mortierellomycotina Class Inc. Sed.	Mortierellaceae							5.6	5.6				
Biocrust	Actinobacteria	Actinobacteria	Geodermatophilaceae	12.7	8.1	13.7	13.7							7.9	7.7
			**Intrasporangiaceae**									8.1	5.6		
			Kineosporiaceae							7.5	5.6				
			**Micrococcaceae**	5.2	5.2	6.6	6.6							6.5	6.2
			**Nocardioidaceae**	29.5	24.9					9.4	9.4			9.8	9.8
		Thermoleophilia	**Gaiellaceae**	4.6	4.6										
	Ascomycota	Dothideomycetes	Mycosphaerellaceae					6.4	4.7						
			**Pleosporaceae**			9.3	8.7	11.6	11.6			11.3	6.3	6.5	4.8
			Pleosporales Family Inc. Sed.	6.9	6.9										
		Lichinomycetes	Lichinaceae							5.6	5.6				
		Sordariomycetes	**Nectriaceae**	9.8	8.1	10.4	8.7	8.7	0.0					6.5	6.5
	Bacteroidetes	Bacteroidia	**Chitinophagaceae**	6.4	0.6	13.1	13.1			19.1	8.8			12.7	5.3
			Microscillaceae					6.4	5.8			8.8	8.8		

Basidio-mycota	Tremello-mycetes	Piskurozymaceae							7.2	7.2				
Cyano-bacteria	Oxyphoto-bacteria	“Unknown Family”	15.6	2.3							6.3	2.5		
Plancto-mycetes	Planctomycetacia	Pirellulaceae					7.0	7.0			11.3	9.4		
Proteo-bacteria	Alpha-proteobacteria	Azospirillaceae											7.4	7.2
		Beijerinckiaceae							6.3	5.6				
		Hyphomonadaceae											5.0	5.0
		Rhizobiaceae			7.1	2.7								
		**Rhodobacteraceae**					5.2	4.7	6.3	5.3	6.3	5.0		
		**Sphingomonadaceae**			22.4	18.6	27.3	17.4	10.6	7.2	26.9	16.3	10.8	7.2
		Xanthobacteraceae					14.0	14.0			8.1	7.5		
	Delta-proteobacteria	Sandaracinaceae									6.3	5.0		
	Gamma-proteobacteria	**Burkholderiaceae**	11.6	11.0	9.3	8.7			7.2	5.9			12.7	10.8
		Rhodano-bacteraceae	5.2	2.3										
		“Unknown Family”					5.8	4.7						
		**Xanthomonadaceae**			7.1	7.1								
Verruco-microbia	Verruco-microbiae	Chthonio-bacteraceae									6.9	5.0		
		Opitutaceae			8.7	6.6			6.6	6.6				
		Pedosphaeraceae					11.1	10.5						

2. Figures

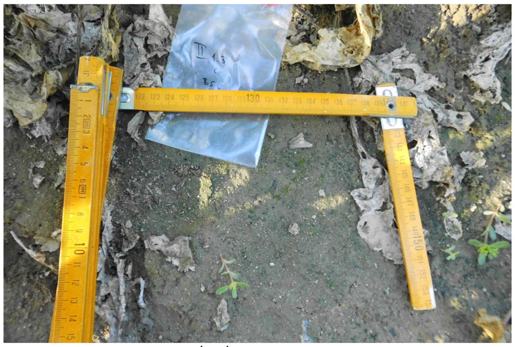

conventional Tillage, 120 kg N a^{-1} ha^{-1}, without crop residues

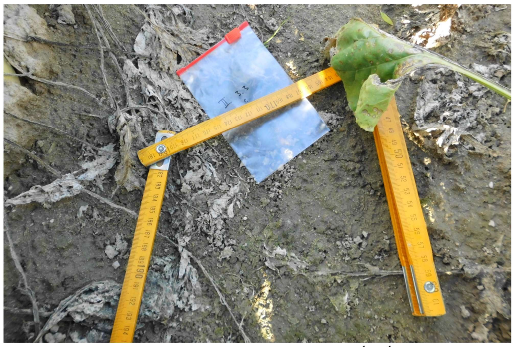

conventional Tillage, 120 kg N a^{-1} ha^{-1}, with crop residues

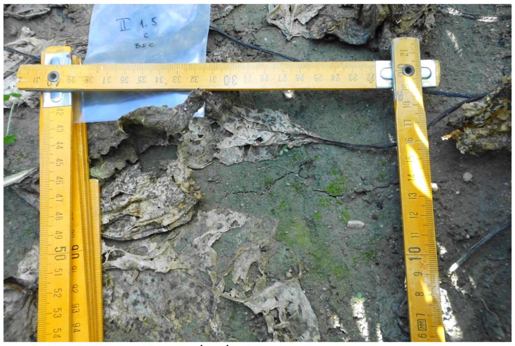

conventional Tillage, 240 kg N a^{-1} ha^{-1}, without crop residues

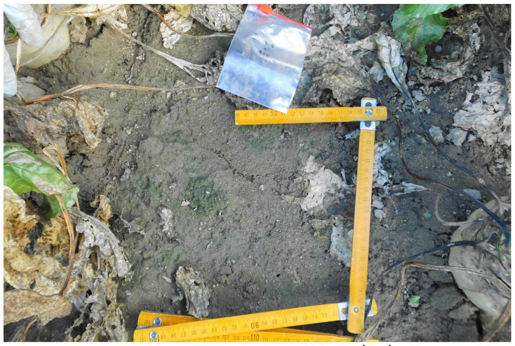

conventional Tillage, 240 kg N a^{-1} ha^{-1}, with crop residues

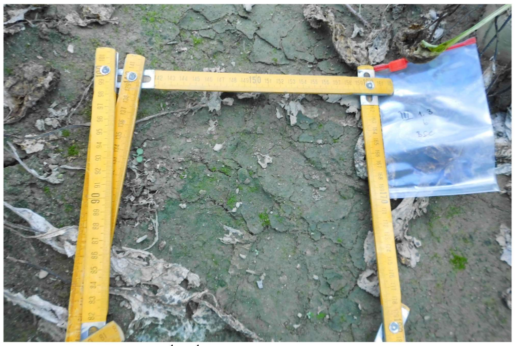

reduced Tillage, 120 kg N a^{-1} ha^{-1}, without crop residues

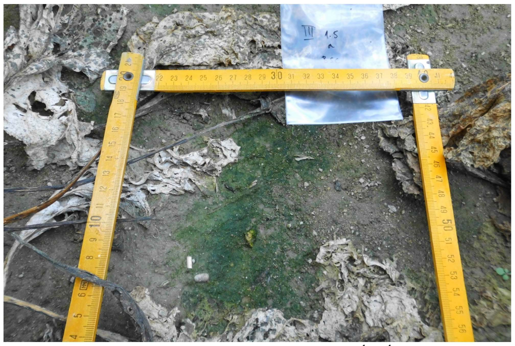

reduced Tillage, 120 kg N a^{-1} ha^{-1}, with crop residues

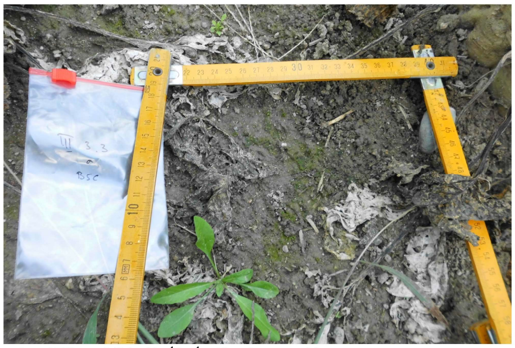

reduced Tillage, 240 kg N a^{-1} ha^{-1}, without crop residues

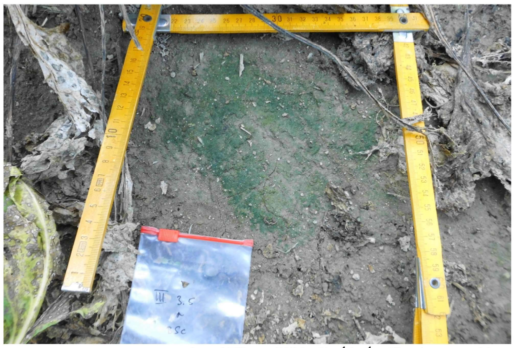

reduced Tillage, 240 kg N a^{-1} ha^{-1}, without crop residues

Fig. S1 Pictures of Biocrusts on the fields of IOSDV LUFA Speyer. One example is shown for each treatment. October 5th, 2016.

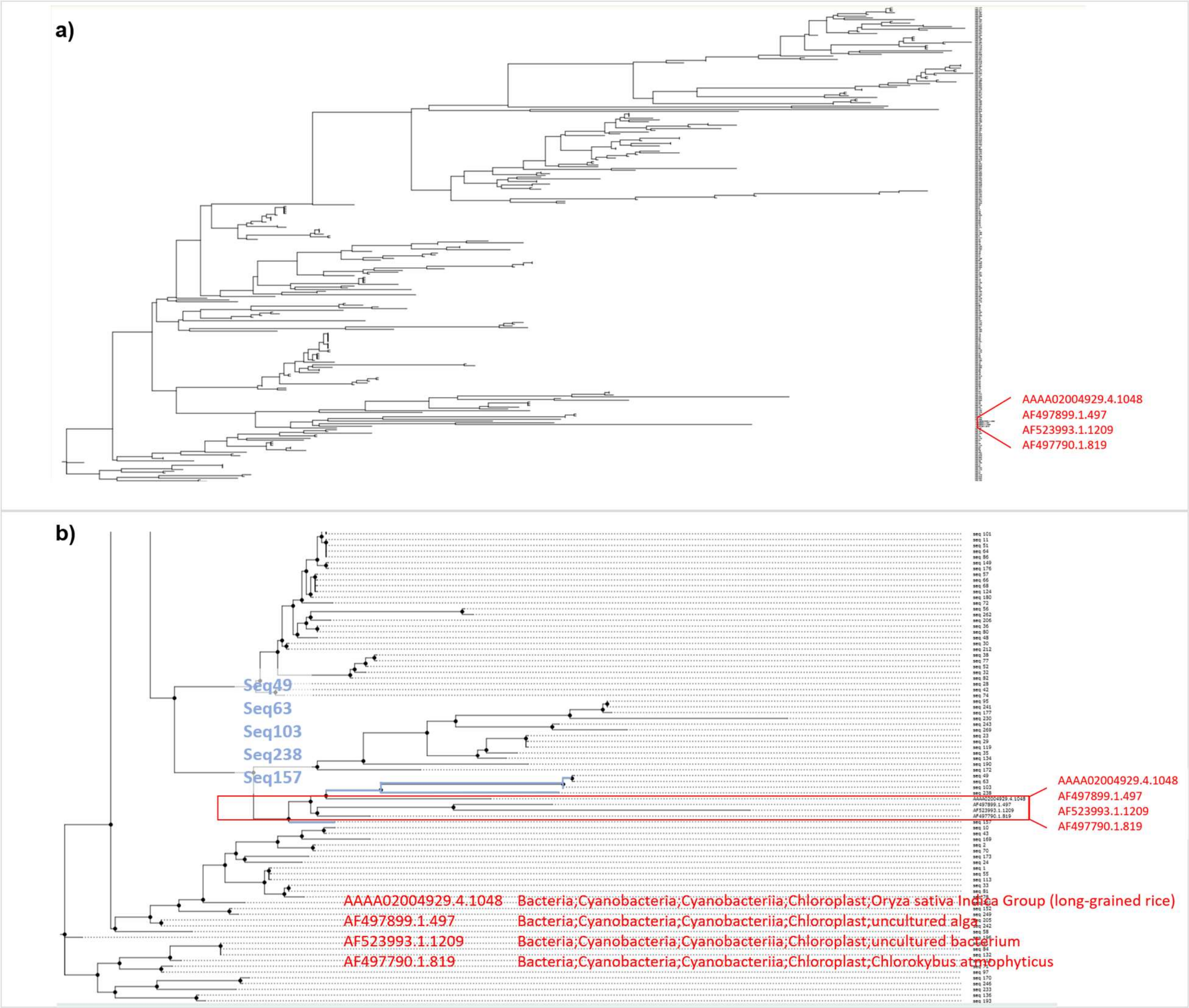

Fig. S2 Phylogenetic tree to exclude those ASVs falsely identified taxonomically as Cyanobacteria but of chloroplast origin. The tree was constructed using the "SILVA Alignment, Classification and Tree (ACT) Service" from the Silva database Version 138.1 (Quast *et al.*, 2013) including all ASVs of our study identified as Cyanobacteria and representative chloroplast reads from plants (AAAA02004929.4.1048 *Oryza sativa Indica Group* (long-grained rice)), Algae (AF497899.1.497 uncultured alga, AF497790.1.819 *Chlorokybus atmophyticus*) and Bacteria (AF523993.1.1209 uncultured bacterium). As shown in sub figure a), chloroplast reads formed a separate cluster. A zoom in sub figure b) shows that branches of five ASVs (Seq49, Seq63, Seq103, Seq238, Seq157) group close to the chloroplast reads but these ASVs display only a maximum of 1.1% of all reads (after subsampling) and were not specified further than to the order level (Bacteria; Cyanobacteria; Oxyphotobacteria; Nostocales; unspec. and Bacteria; Cyanobacteria; Oxyphotobacteria; unspec.) and were thus below the abundance cut-off for further analysis.

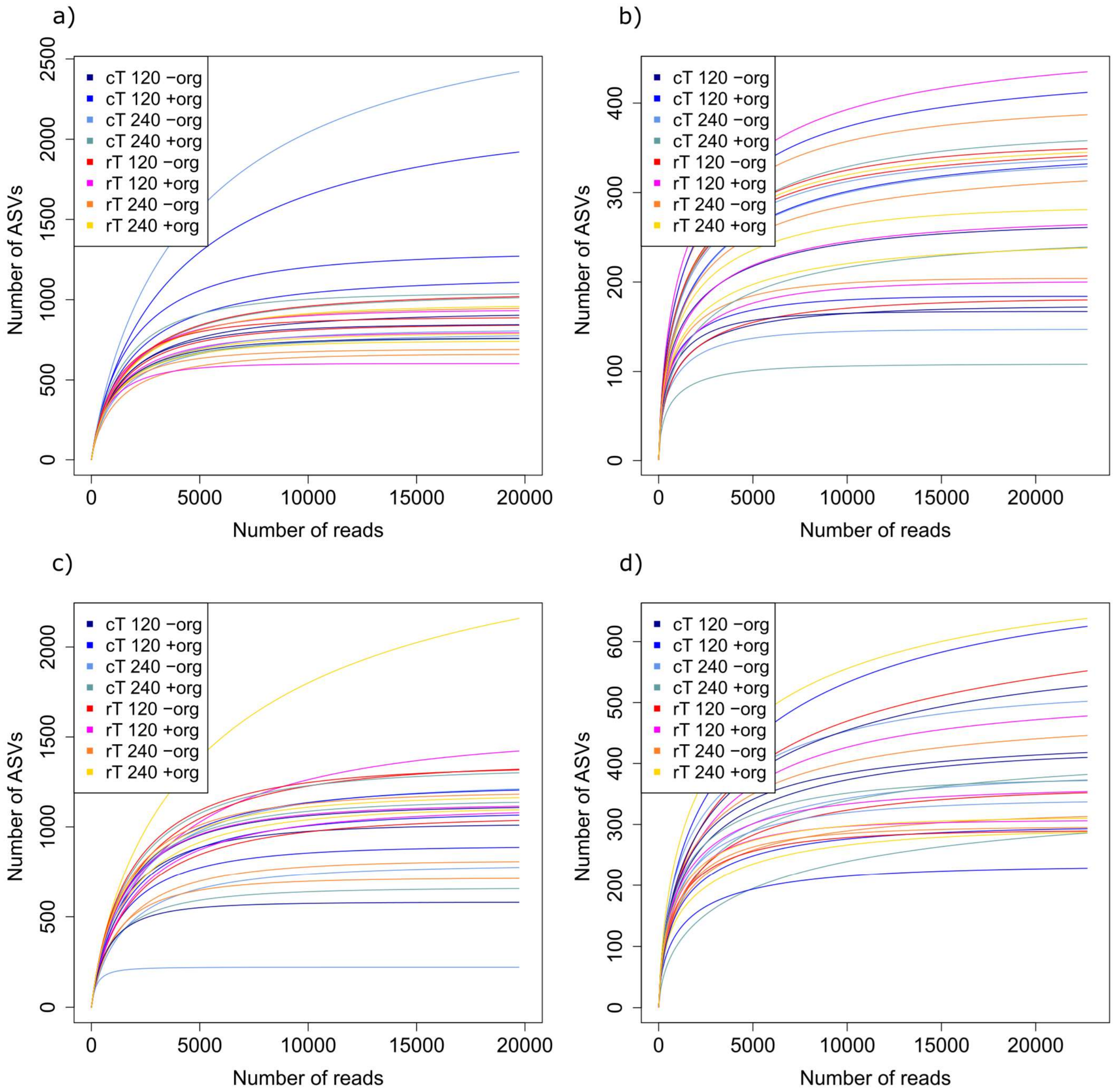

Fig. S3 Rarefaction curves on ASV level for each replicate of a) Biocrust Prokaryotes, b) Biocrust Fungi, c) Bare soil Prokaryotes, d) Bare soil Fungi on subsampled data. Treatments are given for tillage (conventional (cT) vs. reduced tillage (rT)), organic amendments (without (-org) vs. with crop residues (+org)), and mineral fertilization amounts (120 vs. 240 kg N/ha·a).

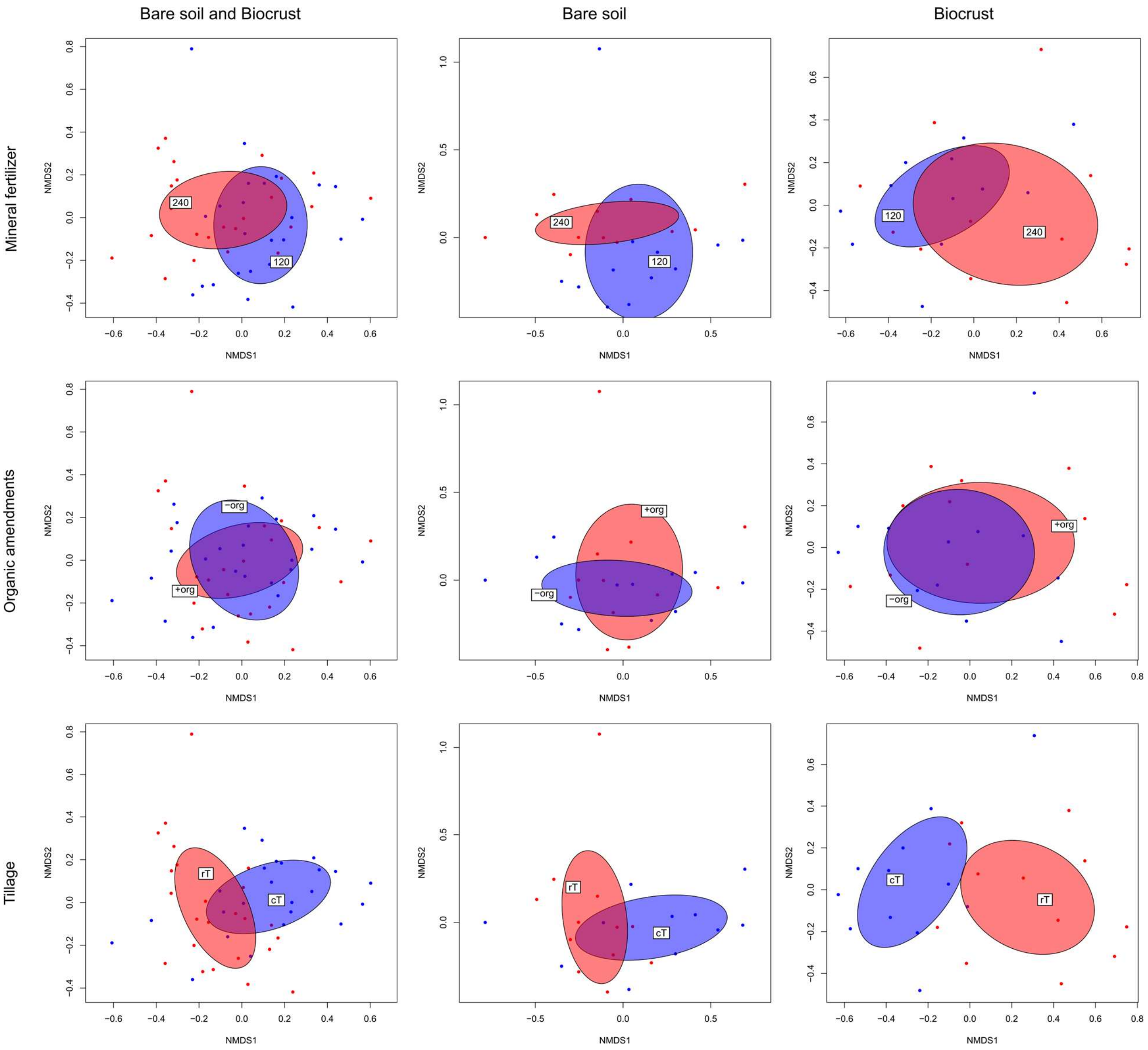

Fig. S4 NMDS plots for prokaryotic community composition at the species level in bare soils and biocrusts colored by treatment (N = 24). Treatments are given for tillage (conventional (cT) vs. reduced tillage (rT)), organic amendments (without (-org) vs. with crop residues (+org)), and mineral fertilization amounts (120 vs. 240 kg N/ha·a).

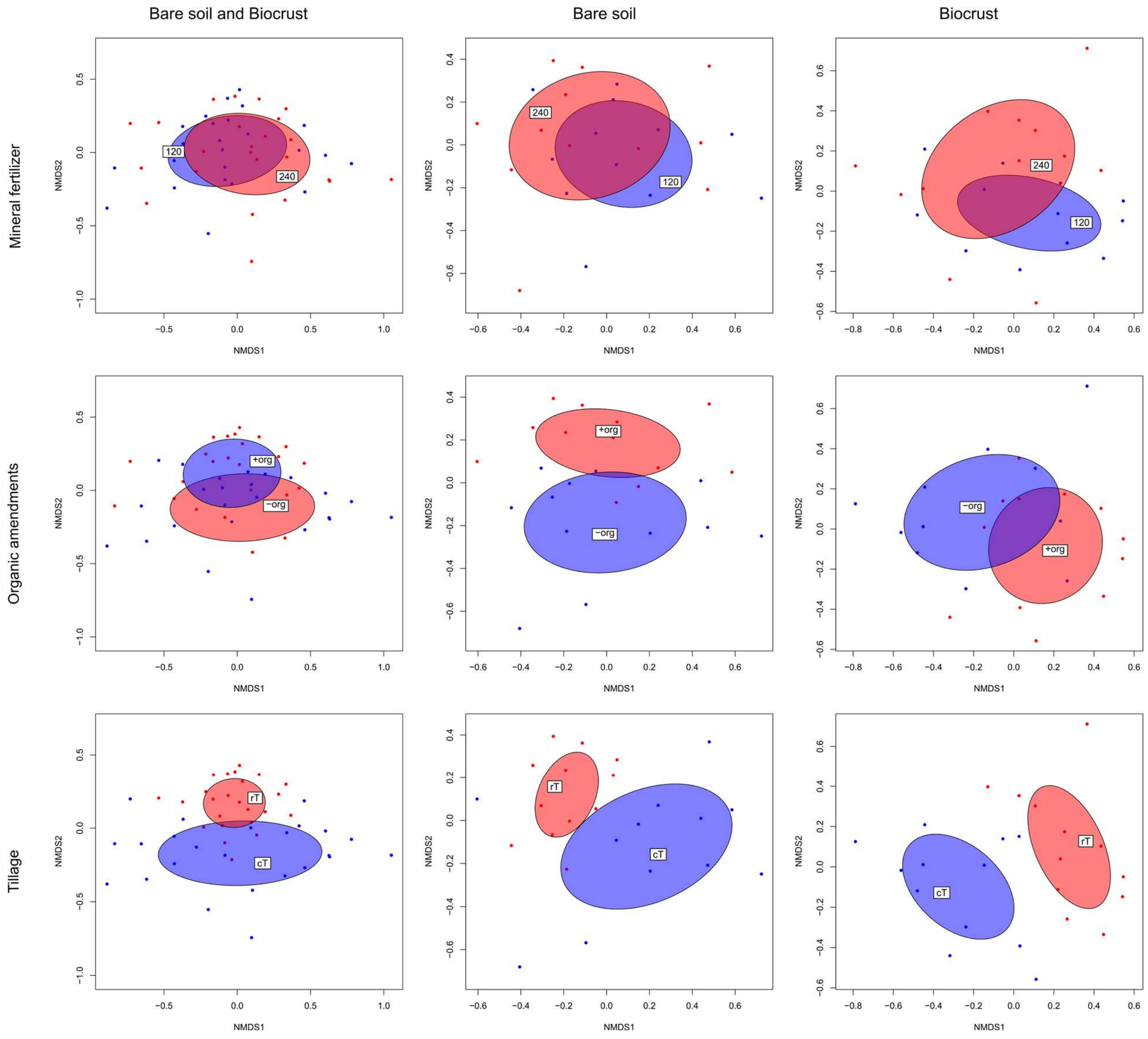

Fig. S5 NMDS plots for fungal community composition at the species level in bare soils and biocrusts colored by treatment (N = 24). Treatments are given for tillage (conventional (cT) vs. reduced tillage (rT)), organic amendments (without (-org) vs. with crop residues (+org)), and mineral fertilization amounts (120 vs. 240 kg N/ha·a).

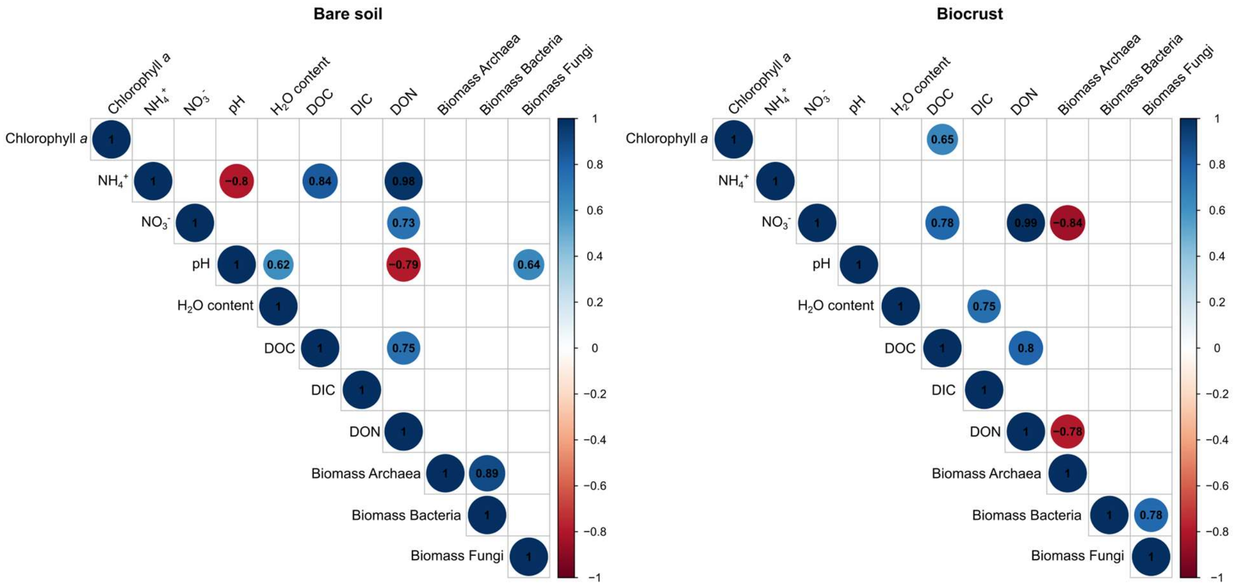

Fig. S6 Significant ($p \leq 0.05$) Pearson correlations ($r > 0.4$ and $r < -0.4$) for bare soils and biocrusts (each N = 24) of soil properties and microbial biomass, where positive correlations ($r > 0$) are shown in blue and negative ones ($r < 0$) are shown in red. R values are given in the circles.

References

Bach, H.J., Tomanova, J., Schloter, M. and Munch, J.C. (2002), “Enumeration of total bacteria and bacteria with genes for proteolytic activity in pure cultures and in environmental samples by quantitative PCR mediated amplification”, *Journal of Microbiological Methods*, Elsevier, Vol. 49 No. 3, pp. 235–245, doi: 10.1016/S0167-7012(01)00370-0.

Bano, N., Ruffin, S., Ransom, B. and Hollibaugh, J.T. (2003), “Phylogenetic Composition of Arctic Ocean Archaeal Assemblages and Comparison with Antarctic Assemblages”, *Applied and Environmental Microbiology*, Vol. 70, No2 No. 2, pp. 781–789, doi: 10.1128/AEM.70.2.781-789.2004.

Herlemann, D.P., Labrenz, M., Jürgens, K., Bertilsson, S., Waniek, J.J. and Andersson, A.F. (2011), “Transitions in bacterial communities along the 2000 km salinity gradient of the Baltic Sea”, *The ISME Journal*, Nature Publishing Group, Vol. 5 No. 10, pp. 1571–1579, doi: 10.1038/ismej.2011.41.

Klindworth, A., Pruesse, E., Schweer, T., Peplies, J., Quast, C., Horn, M. and Glöckner, F.O. (2013), “Evaluation of general 16S ribosomal RNA gene PCR primers for classical and next-generation sequencing-based diversity studies”, *Nucleic Acids Research*, Oxford Academic, Vol. 41 No. 1, pp. e1–e1, doi: 10.1093/nar/gks808.

Nicol, G.W., Glover, L.A. and Prosser, J.I. (2003), “The impact of grassland management on archaeal community structure in upland pasture rhizosphere soil”, *Environmental Microbiology*, John Wiley & Sons, Ltd, Vol. 5 No. 3, pp. 152–162, doi: 10.1046/j.1462-2920.2003.00399.x.

Quast, C., Pruesse, E., Yilmaz, P., Gerken, J., Schweer, T., Yarza, P., Peplies, J., *et al.* (2013), “The SILVA ribosomal RNA gene database project: improved data processing and web-based tools”, *Nucleic Acids Research*, Nucleic Acids Res, Vol. 41 No. D1, doi: 10.1093/NAR/GKS1219.

Tedersoo, L., Anslan, S., Bahram, M., Põlme, S., Riit, T., Liiv, I., Kõljalg, U., *et al.* (2015), “Shotgun metagenomes and multiple primer pair-barcode combinations of amplicons reveal biases in metabarcoding analyses of fungi”, *MycoKeys*, Pensoft Publishers, Vol. 10, pp. 1–43, doi: 10.3897/mycokeys.10.4852.

White, T.J., Bruns, T.D., Lee, S.B. and Taylor., J.W. (1990), “Amplification and direct sequencing of fungal ribosomal RNA Genes for phylogenetics”, *PCR Protocols: A Guide to Methods and Applications*, doi: 10.1016/b978-0-12-372180-8.50042-1.
